# Development of an Amplicon-Based Next-Generation Sequencing Protocol to Identify *Leishmania* Species and Other Trypanosomatids in Leishmaniasis Endemic Areas

**DOI:** 10.1128/Spectrum.00652-21

**Published:** 2021-10-13

**Authors:** Luz H. Patiño, Adriana C. Castillo-Castañeda, Marina Muñoz, Jesus E. Jaimes, Nicolas Luna-Niño, Carolina Hernández, Martha S. Ayala, Patricia Fuya, Claudia Mendez, Carlos E. Hernández-Pereira, Lourdes Delgado, Claudia M. Sandoval-Ramírez, Plutarco Urbano, Alberto Paniz-Mondolfi, Juan David Ramírez

**Affiliations:** a Centro de Investigaciones en Microbiología y Biotecnología-UR (CIMBIUR), Facultad de Ciencias Naturales, Universidad del Rosario, Bogotá, Colombia; b Grupo de Parasitología, Instituto Nacional de Salud, Bogotá, Colombia; c Grupo de Investigación en Enfermedades Tropicales del Ejército (GINETEJ), Laboratorio de Referencia e Investigación, Dirección de Sanidad Ejército, Bogotá, Colombia; d Instituto de Investigaciones Biomédicas IDB/Incubadora Venezolana de la Ciencia, Barquisimeto, Venezuela; e Grupo de Investigaciones en Ciencias Básicas y Aplicadas para la Sostenibilidad (CIBAS), Facultad de Ciencias Exactas, Naturales y Agropecuarias, Universidad de Santander, Bucaramanga, Colombia; f Grupo de Investigaciones Biológicas de la Orinoquia, Universidad Internacional del Trópico Americano (Unitropico), Yopal, Colombia; g Icahn School of Medicine at Mount Sinai, New York, New York, USA; Dublin City University

**Keywords:** amplicon-based NGS, *Leishmania*, NGS, species

## Abstract

Trypanosomatid infections are an important public health threat affecting many low-income countries across the tropics, particularly in the Americas. Trypanosomatids can infect many vertebrate, invertebrate, and plant species and play an important role as human pathogens. Among these clinically relevant pathogens are species from the genera *Leishmania* and *Trypanosoma*. Mixed trypanosomatid infections remain a largely unexplored phenomenon. Herein, we describe the application of an amplicon-based next-generation sequencing (NGS) assay to detect and identify trypanosomatid species in mammalian reservoirs, human patients, and sand fly vectors throughout regions of *Leishmania* endemicity. Sixty-five samples from different departments of Colombia, including two samples from Venezuela, were analyzed: 49 samples from cutaneous leishmaniasis (CL) patients, 8 from sand flies, 2 from domestic reservoirs (Canis familiaris), and 6 from wild reservoirs (Phyllostomus hastatus). DNA from each sample served to identify the presence of trypanosomatids through conventional PCR using heat shock protein 70 (HSP70) gene as the target. PCR products underwent sequencing by Sanger sequencing and NGS, and trypanosomatid species were identified by using BLASTn against a reference database built from trypanosomatid-derived HSP70 sequences. The alpha and beta diversity indexes of amplicon sequence variants were calculated for each group. The results revealed the presence of mixed infections with more than two *Leishmania* species in 34% of CL samples analyzed. Trypanosoma cruzi was identified in samples from wild reservoirs, as well as in sand fly vectors. Coinfection events with three different *Leishmania* species were identified in domestic reservoirs. These findings depose the traditional paradigm of leishmaniasis as being a single-species-driven infection and redraw the choreography of host-pathogen interaction in the context of multiparasitism. Further research is needed to decipher how coinfections may influence disease progression. This knowledge is key to developing an integrated approach for diagnosis and treatment.

**IMPORTANCE** Traditionally, there has been a frequent, yet incorrect assumption that phlebotomine vectors, animal reservoirs, and human hosts are susceptible to *Leishmania* infection by a single parasite species. However, current evidence supports that these new vector-parasite-reservoir associations lend vectors and reservoirs greater permissiveness to certain *Leishmania* species, thus promoting the appearance of coinfection events, particularly in disease-endemic regions. The present study describes the application of an amplicon-based next-generation sequencing (NGS) assay to detect and identify trypanosomatid species in mammalian reservoirs, human patients, and sand fly vectors from regions of endemicity for leishmaniasis. This changes our understanding of the clinical course of leishmaniasis in areas of endemicity.

## INTRODUCTION

Leishmaniasis is a complex vector-borne infectious disease caused by parasites of the genus *Leishmania*, which are transmitted by insect vectors of the Psychodidae family. The disease exhibits a wide clinical spectrum, driven not only by the host’s immune response but also by the infecting parasite species. To date, three main clinical forms of the disease are recognized, cutaneous leishmaniasis (CL), visceral leishmaniasis (VL), and mucocutaneous leishmaniasis (MCL), with CL being the mildest and most common form of the disease. According to WHO, about 1.2 million CL cases have been reported worldwide, with nearly 75% of them occurring in 10 countries: Afghanistan, Algeria, Brazil, Iran, Syria, Ethiopia, North Sudan, Costa Rica, Peru, and Colombia (1).

Current knowledge suggests that different demographic and geographical factors may promote substantial variation in the ecoepidemiological patterns associated with *Leishmania* transmission cycles. Such variations not only reflect the increased spatial distributions and habitat ranges of different sand fly vector species (2) and reservoirs (3) but also appear to influence the emergence of new vector-parasite-reservoir associations (4).

Traditionally, there has been a frequent yet incorrect assumption that phlebotomine vectors, animal reservoirs, and human hosts are susceptible to infection by a single parasite species. However, current evidence supports that these new vector-parasite-reservoir associations lend vectors and reservoirs greater permissiveness to certain *Leishmania* species, thus promoting the appearance of coinfection events, particularly in regions where the disease is endemic (5, 6).

So far, several natural human cooccurring infectious events involving diverse *Leishmania* species have been reported worldwide. In Old World countries, such as Sudan and Iran, cases of Leishmania donovani and Leishmania major (CL patients) (7) and Leishmania tropica and L. major (in an MCL patient) (8) coinfection have been documented. Moreover, several coinfections have also been described in the New World. For example, in Bolivia, a case of Leishmania amazonensis and Leishmania infantum (Leishmania chagasi) coinfection was described in a patient with severe diffuse CL (5). In Mexico, Monroy-Ostria et al. reported on a patient with synchronous Leishmania mexicana and Leishmania braziliensis infection (9). Veland et al. also documented a case of simultaneous infection by L. braziliensis and Leishmania lainsoni in a Peruvian patient with CL (6). More interestingly, the presence of Leishmania guyanensis and Leishmania panamensis hybrid strains causing both CL and MCL has been described in Ecuador (10). Furthermore, studies from Brazil have revealed the presence of double and triple infections caused by several *Leishmania* species, both in mammals and humans (11, 12).

Likewise, natural coinfections in sand fly vectors and reservoirs have been reported, as in the case of dogs (Canis lupus familiaris) and rodents (Mus musculus and Rattus rattus) infected with L. braziliensis and L. infantum from Brazil (13) or the case of naturally occurring *Leishmania* and *Trypanosoma* coinfections observed across different genera of sand flies and mammals (11, 14, 15). Despite the well-documented occurrence of coinfection events among mammalian reservoirs and sand fly vectors in the Americas, the significance of this phenomenon remains largely unexplored.

To date, the most common molecular approaches to detect coinfection events in both *Leishmania*-infected vectors and mammalian reservoirs are PCR targeting the kinetoplast DNA (kDNA) (6) or small-subunit (SSU) rRNA gene (13), PCR-restriction fragment length polymorphism (PCR-RFLP) (11, 16), and species-specific PCR. Although fast and sensitive, these methods are often expensive and time consuming, particularly when screening a large number of specimens. On the other hand, the targeted nature of species-specific PCR further precludes recognition of rare and new species, as well as the detection of multiple coamplified genetic variants, thus obscuring the presence of lower-abundance genotypes in the context of coinfections (17).

Next-generation sequencing (NGS), particularly amplicon-based sequencing, has emerged as an alternative option to circumvent such limitations. Various studies capitalizing on the advantages of this technology have been conducted for different purposes, for example, determining the prevalence and diversity of bacterial communities (18, 19) and intestinal protozoa (*Blastocystis*) (20) or characterizing single-nucleotide polymorphisms in viruses (21). Moreover, NGS has allowed the identification of blood-feeding sources of triatomines (22), detection of *Leishmania* parasites, blood sources, and plant meals, and deciphering of the intestinal microbiome of phlebotomine sand flies (23), in addition to assessment of the genetic diversity of trypanosomatids in humans (Trypanosoma cruzi and Trypanosoma rangeli) (24, 25) and mammalian reservoir hosts (17, 26, 27). However, relatively few studies have applied this technique to identify and detect coinfection events associated with *Leishmania* in different biological samples (28).

Here, we designed and implemented a novel amplicon-based NGS assay targeting heat shock protein 70 (HSP70) in order to detect and identify trypanosomatid species infecting not only humans but also mammalian reservoirs and sand fly vectors collected across different geographic regions of endemicity in Colombia and Venezuela. Identifying coinfection events can improve our understanding of pathogen-host interactions and their influence on the epidemiology, pathophysiology, and clinical course of the disease and, additionally, assist in diagnosis and guidance for appropriate therapeutic interventions.

## RESULTS

### HSP70 gene is useful to identify trypanosomatid species.

The specificity of the HSP70 primers used in this study, both intra- and interspecies, was evaluated. At the intraspecies level, the results obtained showed that, in the evaluated species, the primers only annealed in the different copies of the HSP70 gene of the same chromosome. Additionally, it was observed that those sequences recognized by the primers were totally identical to each other (percent identity, 100%), demonstrating that there was no loss of identity between copies in the different chromosomes of the same species. Regarding the interspecies analysis, we performed a phylogenetic analysis with those copies of the HSP70 gene recognized by the primers for each species, and the results demonstrated the high discriminatory power of the primers across the different species evaluated (Fig. S1 in the supplemental material).

**FIG 1:**
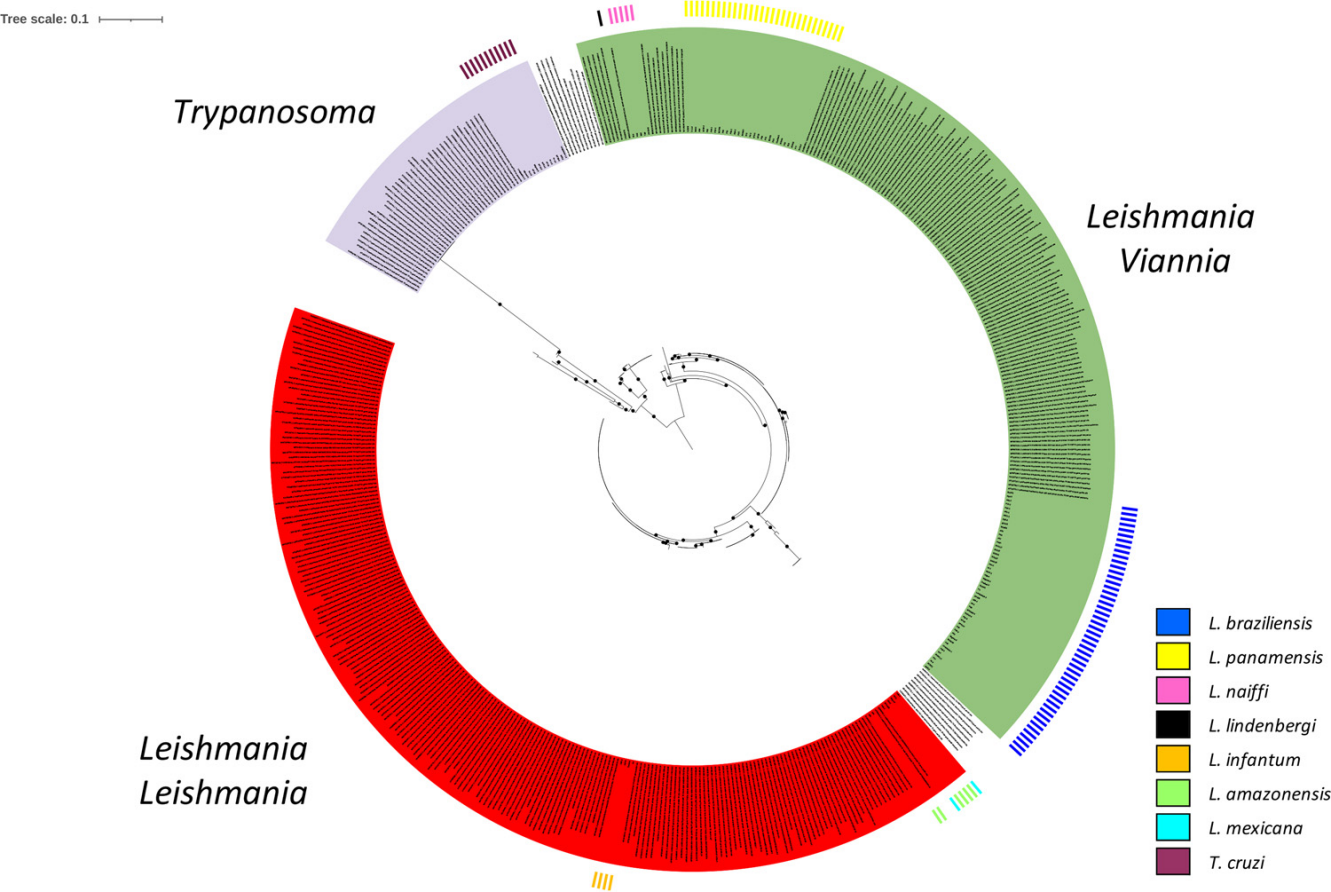
Phylogenetic relationship among the HSP70 sequences used for the reference database and the HSP70 sequences analyzed in this study. The tree represents the phylogenetic analysis based on 385 HSP70 sequences used for the reference database and the 113 HSP70 sequences obtained from 65 samples analyzed in this study. The colors of the inner circle represent the genera, and the outside colors represent each of the species identified in the 113 sequences obtained in this study. The black dots in the tree represent well-supported nodes (bootstrap support of ≥90).

**FIG 2:**
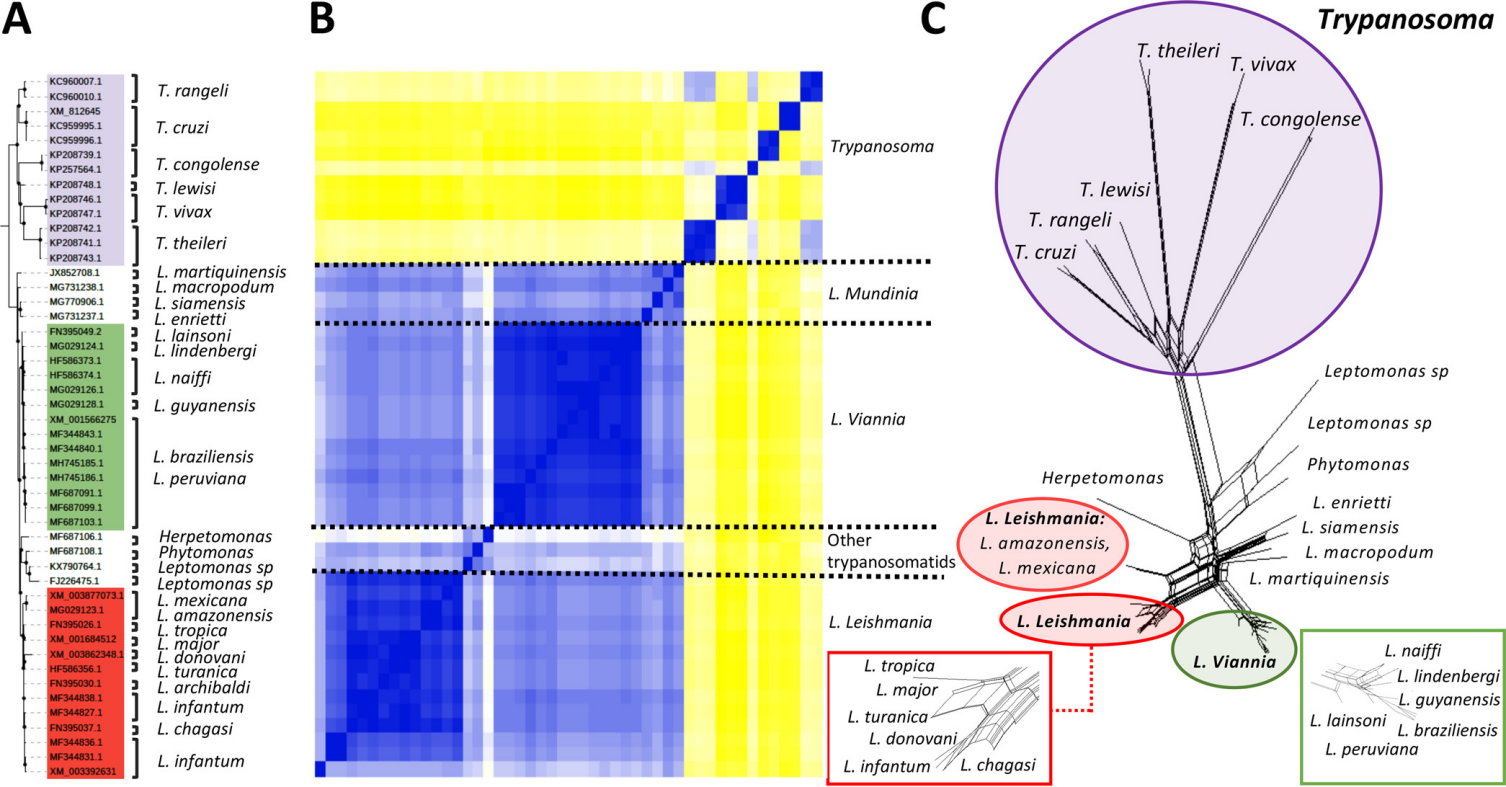
Phylogenetic relationships between the haplotypes from HSP70 sequences used for the reference database. (A) Phylogenetic analysis based on 48 haplotypes from HSP70 reference sequences. The species are listed on the right; the black dots represent well-supported nodes (bootstrap support of ≥90). (B) The heatmap represents the pairwise comparison of the 48 reconstructed haplotypes. The subgenera are listed on the right. (C) Phylogenetic network (Neighbor-Net) constructed in SplitsTree 5. The squares show enlargements of the clusters represented by *Viannia* and *Leishmania* subgenera.

Once the specificity of the HSP70 primers was confirmed, the next step was to determine their ability to identify different species of trypanosomatids. The 385 sequences that constituted the database built as a reference, the 113 sequences from 65 samples included in this study, and the 48 haplotypes were used. The results obtained from the phylogenetic analysis showed, both for the complete data set (385 and 113 sequences) and for the haplotypes, the presence of two populations grouped in well-supported nodes (bootstrap support of >90%) (Fig. 1 and 2A). One was represented by species belonging to the genus *Trypanosoma* (Fig. 1 and 2, highlighted in purple) and the second by different species of the genus *Leishmania*. Within the genus *Leishmania*, the tree topology showed a clear distinction between species of the subgenus *Viannia* (Fig. 1 and 2, highlighted in green) and *Leishmania* species (Fig. 1 and 2, highlighted in red). Four groups were recognized within the *Viannia* subgenus: L. lainsoni, Leishmania naiffi, the L. guyanensis complex, and the L. braziliensis complex. Regarding the *Leishmania* subgenus, three groups were identified, one of them represented by the L. mexicana complex, the second by sequences of L. major and the L. donovani and L. tropica complex, and the third by L. infantum (L. chagasi). These findings were confirmed by analyzing the paired comparison between the haplotypes (Fig. 2B) and supported by the tree topology obtained in SplitsTree5, where the members of the groups were consistently grouped (Fig. 2C). Additionally, the results obtained evidenced correct clustering among the 113 sequences analyzed in this study and the sequences from the reference database (Fig. 1). Finally, we evaluated and compared the nucleotide diversity between the sequences used as reference sequences; the results identified 132 variable sites among the 337 bp that constitute the HSP70 gene fragment, which represents 39% variability among the sequences.

### Identification of *Leishmania* species by Sanger sequencing.

An analysis from CL patients’ samples revealed that the most commonly identified *Leishmania* species was L. braziliensis (63%), followed by L. panamensis (29%), L. amazonensis (4%), L. naiffi (2%), and Leishmania lindenbergi (2%). In domestic reservoirs (Canis familiaris), we identified infection by L. braziliensis (50%) and L. panamensis (50%). Finally, samples from sand fly vectors and wild reservoirs (Phyllostomus hastatus) revealed the presence of L. braziliensis (100%) and T. cruzi (100%), respectively. In none of the samples analyzed the presence of more than one species was identified, (Table S3).

### Sequence analysis by the HSP70 amplicon-based NGS.

A minimum of 134,316 and a maximum of 179,347 paired-end reads were generated after performing the Illumina sequencing of HSP70 amplicons. Subsequently, we analyzed the quality of the reads obtained, observing that in the samples from patients with CL, between 84 and 95% of the reads passed the quality filter (minimal average quality score of 20). In the vectors, the percentages were between 84 and 94%, except for the R95 sample (domestic reservoir), where only 41% of reads passed the control, and in the reservoirs, the range was between 90 and 95%. Finally, we evaluated the number of high-quality reads that were taxonomically assigned by BLASTn. In the samples from patients with CL, we observed that between 71 and 100% of the reads were assigned, in vectors between 60 and 100% were assigned, and finally, in the reservoirs, assignments were made in 88 to 97% of the reads (Fig. S2 and Table S4).

### Method comparisons.

Once we determined that the results obtained for HSP70 amplicon-based sequencing matched those obtained from Sanger sequencing, we performed an agreement analysis between the two methods. The results demonstrated a global agreement of 53% and agreement ranging from 76.9% to 100% with a Kappa coefficient between 0.31 and 1.0 (Fig. S3) when comparing identified species. We hypothesize that the lower global agreement could be due to multispecies identification in the same samples.

### Detection of mixed infections using HSP70 amplicon-based NGS.

**FIG 3:**
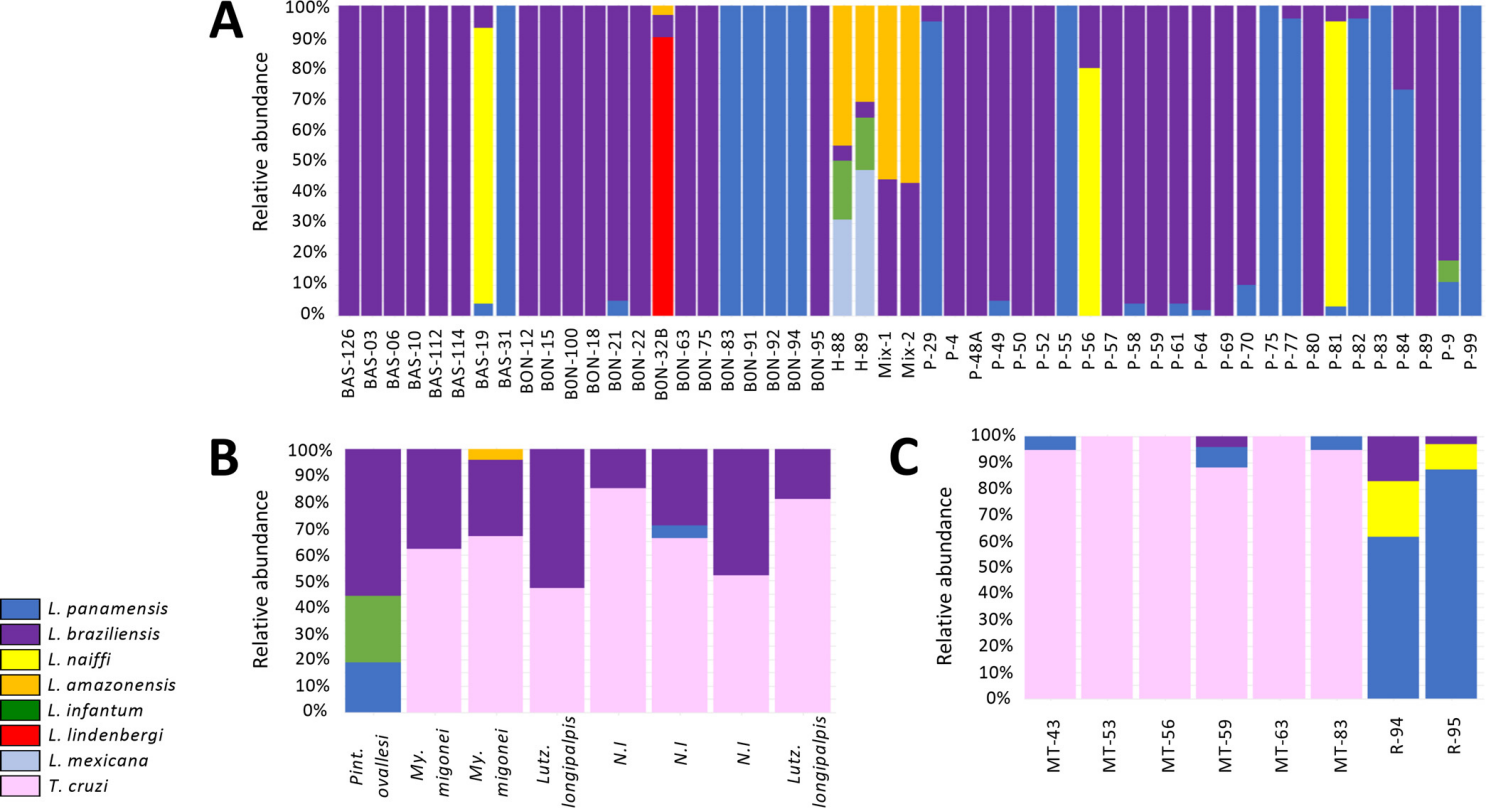
Relative abundances of species identified using the novel amplicon-based NGS assay. The figure represents the relative abundances of *Leishmania* and *Trypanosoma* species found in CL patients (A), sand fly vectors (B), and reservoirs (C). The mixtures of L. braziliensis and L. amazonensis made in the laboratory are identified as mix-1 (1 × 10^6^) and mix-2 (1 × 10^3^). The colors in the bars represent the *Leishmania* and *Trypanosoma* species found.

To determine the capacity of HSP70 amplicon-based NGS, discriminate mixed infections, and estimate the limit of detection of the method, we mixed (1/1) L. braziliensis and L. amazonensis promastigotes at different concentrations in the laboratory and included concentrations of 1 × 10^6^ (mix-1) and 1 × 10^3^ (mix-2) in the analysis. The results obtained demonstrate detection of L. braziliensis and L. amazonensis in the analyzed samples at concentrations proportional to those expected. Additionally, we observed that detection of the relative abundance was maintained at a concentration of up to 10 parasites/sample (Fig. 3A).

### Frequency and diversity of *Leishmania* species in CL patients using amplicon-based NGS.

**FIG 4:**
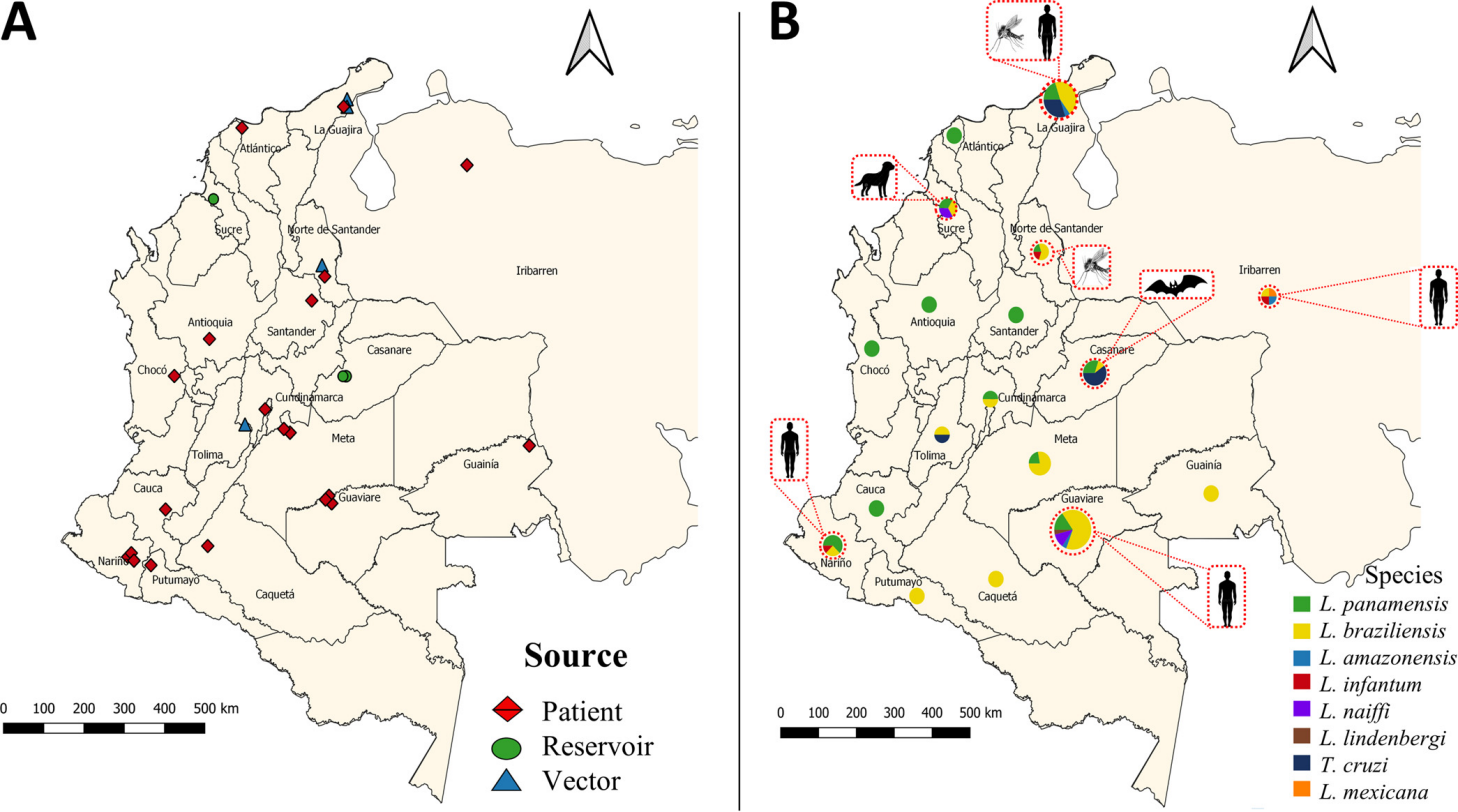
Geographical distribution of samples analyzed in the study and of the species identified through HSP70 amplicon-based NGS. GPS coordinates were used to build georeferenced maps of sampled locations. (A) Geographical localization of 65 samples included in the study. (B) Relative abundances of *Leishmania* and *Trypanosoma* species found in each Colombian department and one state of Venezuela (Lara). The size of the circle refers to the number of samples collected by the department.

The NGS analysis revealed infection and coinfection events in CL patient samples. Sixty-six percent of samples revealed single-species infection, with L. braziliensis being the predominant species (72%), followed by L. panamensis (28%). An interesting finding was the identification of L. mexicana and L. infantum in 4% and 6% of the samples analyzed, previously not identified by Sanger sequencing (Fig. 3A). Additionally, multiple coinfection events were recorded in 34% of samples, which were also not visualized through Sanger sequencing. In these coinfection events, the frequency of infection by L. braziliensis was 80%, followed by L. panamensis in 42% of cases and L. mexicana in 4%. L. amazonensis, L. naiffi, and L. infantum were each present in 6% of cases. L. lindenbergi was present in 2% (Fig. 3A). Double infections were detected in 11 samples, 9 of them, from the departments of Meta, Guaviare, Nariño, and Cundinamarca, showing L. braziliensis/L. panamensis coinfection. Two samples from Guaviare revealed coinfections with L. naiffi/L. panamensis and L. naiffi/L. braziliensis (L. naiffi with a significant prevalence). Triple coinfections (L. lindenbergi/L. braziliensis/L. amazonensis, L. braziliensis/L. panamensis/L. infantum, and L. naiffi/L. braziliensis/L. panamensis) were recorded in three samples from Nariño and Guaviare, and finally, two samples from Venezuela presented multiple coinfection with four *Leishmania* species (L. amazonensis/L. mexicana/L. infantum/L. braziliensis) (Fig. 3A and 4B).

### Frequency and diversity of trypanosomatids in sand fly vectors and reservoirs using amplicon-based NGS.

Coinfection events with two or more *Leishmania* species and/or with Trypanosoma cruzi were observed in sand fly vectors and reservoirs. The results obtained from sand flies allowed us to identify coinfection events in all specimens analyzed. A remarkably high proportion of the reads (>60%) identified T. cruzi (present in 87% of all vectors). For the remaining samples, reads from three different *Leishmania* species (L. panamensis, L. braziliensis, and L. infantum) were recorded (Fig. 3B). The frequency of infection with T. cruzi and L. braziliensis was 87.5% for each, followed by L. panamensis with 25% and L. amazonensis and L. infantum with 12.5% each.

On the other hand, we observed the presence of T. cruzi in all samples from wild reservoirs (Phyllostomus hastatus), where 50% of samples evidenced T. cruzi at 100% of reads, while the remaining 50% evidenced low proportions of reads (between 4 and 8%) of L. panamensis and L. braziliensis, with infection frequencies of 50% and 16%, respectively (Fig. 3C). Regarding domestic reservoirs (Canis familiaris), we observed coinfection events with three different *Leishmania* species, L. braziliensis/L. panamensis/L. naiffi, in the two samples analyzed, with a frequency of infection of 100% for each species.

### Statistical analysis.

The nonparametric Mann-Whitney-Wilcoxon test was performed to explore potential differences between single and coinfection groups, and the Kruskal-Wallis test was applied to explore the differences between the several coinfection groups. The results did not reveal differences related to the continuous variables (age, disease evolution in months, and number of lesions) between single and coinfection groups (Mann-Whitney-Wilcoxon test, with *P* values of 0.08 for age, 0.87 for disease evolution in months, and 0.437 for number of lesions) or between the coinfection groups (Kruskal-Wallis test, *P* values of 0.23 for age, 0.181 for disease evolution in months, and 0.617 for number of lesions).

Chi-square tests and Fisher exact tests were performed to identify potential associations between the categorical variables and species groups. The results showed a relationship between the departments evaluated and the species identified (chi-square test, *P* = 0.03); however, *post hoc* comparisons did not have sufficient statistical power due to low or no availability of data for certain departments. In a descriptive approach to reveal the potential differences between the presence of species in the different departments, it was found that in the department of Guaviare, where the largest proportion of samples were obtained (37.5%), L. braziliensis was the most predominant species (66.6%), followed by L. braziliensis/L. panamensis (11.1%) L. naiffi/L. braziliensis (5.5%), L. naiffi/L. panamensis (5.5%) L. lindenbergi/L. braziliensis/L. amazonensis (5.5%), and L. naiffi/L. braziliensis/L. panamensis (5.5%). In Atlantico, Antioquia, Cauca, Choco, Nariño, and Santander, we identified L. panamensis. In Caqueta, Guanía, Meta, Norte de Santander, and Putumayo, we found only L. braziliensis (Fig. 4B and Table S5). We did not identify statistical differences between biological, ecological, and epidemiological variables and species groups in sand fly vectors and reservoirs.

### Diversity analysis.

The alpha diversity of amplicon sequence variants (ASVs) (Simpson index [dominance] and Shannon index [diversity]) revealed statistically significant differences between the groups analyzed (Kruskal-Wallis test, *P* = 1.734e−06 and *P* = 1.979e−06, respectively). We performed a pairwise *post hoc* comparison based on the Dunn-Bonferroni test to determine the groups among which these differences were present.

**FIG 5:**
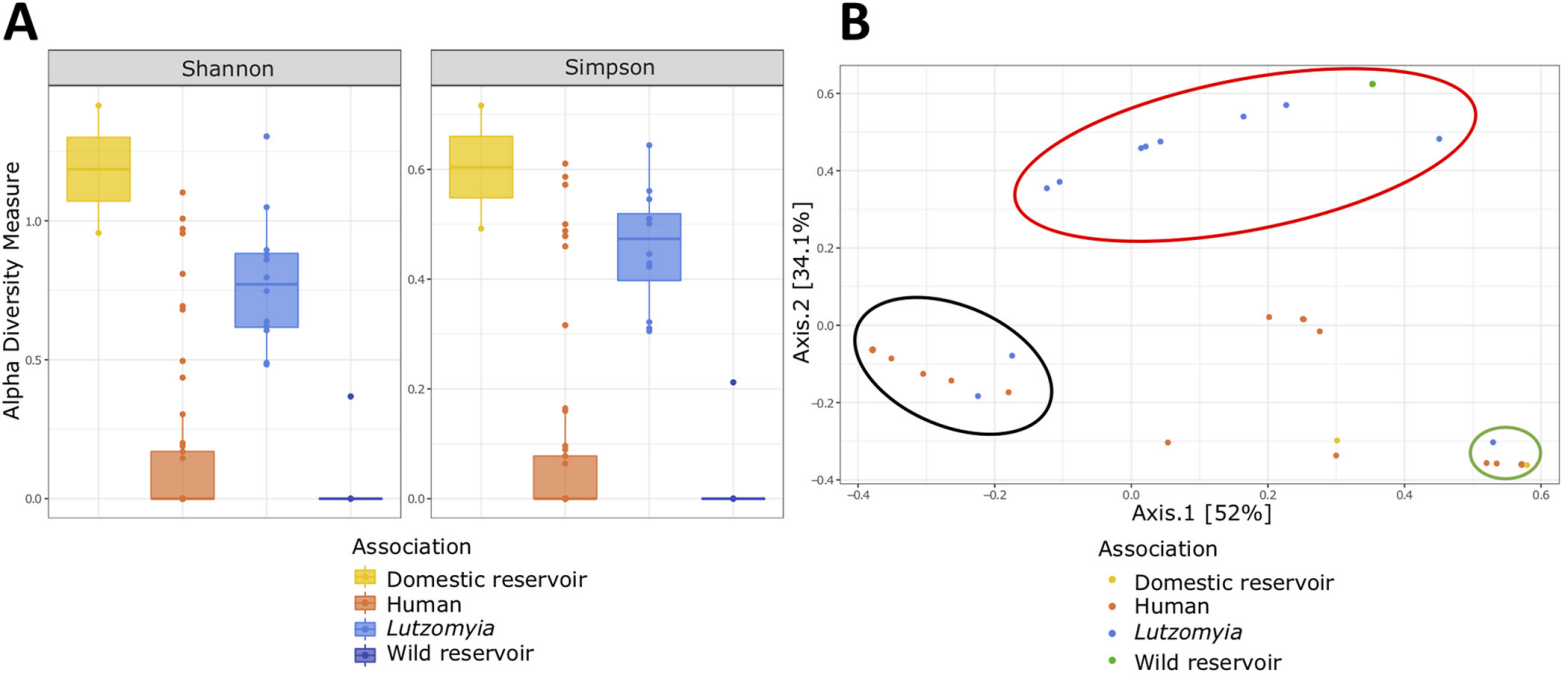
Sequence diversity analysis of HSP70 reads. (A) Boxplots displaying Shannon and Simpson index results for each group evaluated (human CL, *Lutzomyia*, and wild and domestic reservoirs). (B) Principal coordinate analysis (PCoA) plots between the groups evaluated. The ovals encompass those individuals that had the same diversity of species of trypanosomatids.

Domestic reservoirs and sand flies exhibited higher diversity (Shannon) indices of 1.3 and 0.75, respectively, than did humans and wild reservoirs, where the median values obtained for both were close to 0 (Kruskal-Wallis test, *P* = 1.979e−06, and *post hoc* Dunn-Bonferroni, *P* = 0.031 and *P* = 0.002, respectively) (Fig. 5A). Correspondingly, the dominance (Simpson) indices were higher in domestic reservoirs and sand flies (0.6 and 0.48, respectively) than in humans and wild reservoirs (Kruskal-Wallis test, *P* = 1.734e−06, and *post hoc* Dunn-Bonferroni, *P* = 0.00001 and *P* = 0.0021, respectively) (Fig. 5A). Regarding pairwise comparisons among groups, the Shannon index analysis revealed statistically significant differences between domestic reservoirs and humans, sand flies and humans, domestic reservoirs and wild reservoirs, and sand flies and wild reservoirs (Table S6); however, the Simpson index analysis revealed statistically significant differences only between humans and sand flies and between sand flies and wild reservoirs (Table S6).

Permutational multivariate analysis of variance (PERMANOVA) showed statistically significant differences of beta diversity among the populations analyzed (PERMANOVA, *P* < 0.05). Similarity percentage (SIMPER) analysis revealed that ASVs corresponding to L. panamensis, L. braziliensis, and T. cruzi contributed significantly to the dissimilarity among clustering. Three groups were observed; the first comprised 15 human and 2 sand fly samples (Fig. 5B), where L. braziliensis (PERMANOVA, *P* = 0.0094) and T. cruzi (PERMANOVA, *P* = 0.0002) were the genera that mainly contributed to the differentiation, with contributions of 35.84 and 36.82%, respectively (Table S7). The second group included all the wild reservoir samples and nine sand fly samples (Fig. 5B). However, the results revealed that none of the ASVs identified showed statistically significant differences, indicating that they did not contribute to the differentiation of the grouping (PERMANOVA, *P* > 0.05) (Table S7). Finally, the last group was represented by human, sand fly, and domestic reservoir samples, where L. panamensis, L. braziliensis, and T. cruzi contributed to the dissimilarity. L. braziliensis (PERMANOVA, *P* = 00026) and L. panamensis (PERMANOVA, *P* = 0.048) were the *Leishmania* species that mostly contributed to the differentiation between humans and domestic reservoirs, with contributions of 43 and 41.57%, respectively, and L. panamensis (PERMANOVA, *P* = 0.038) and T. cruzi (PERMANOVA, *P* = 0.042) were the genera that contributed the most to the differentiation between sand flies and domestic reservoirs, with contributions of 38.63 and 33.63%, respectively (Table S7).

## DISCUSSION

Mixed trypanosomatid infections remain a largely unexplored phenomenon. However, given the marked heterogeneity in ecoepidemiological patterns associated with trypanosomatid transmission and the ever-increasing trends in spatial distribution of different vector species (2) and reservoirs, it seems reasonable to infer that the prevalence of trypanosomatid polyparasitism may be higher than previously thought. Deciphering the true prevalence of coinfections would help to improve our understanding of the interactions between these pathogens and the implications for host health.

Herein, we designed an NGS-based method for the detection of mixed trypanosomatid infections in CL patients (Fig. 3A) in order to assess the prevalence and genetic diversity not only in human hosts but also in sand fly vectors and mammalian reservoirs. Our data analysis revealed that the most commonly observed coinfection events were caused by L. braziliensis/L. panamensis (Fig. 3A and 4B). This was predictable given the high prevalence and overlapping occurrence of these species across most regions of endemicity in Colombia (29, 30).

Currently, little is known about the effects of coinfecting pathogen interactions and how this may influence disease outcomes. Previous studies have suggested that preceding *Leishmania* infections may confer protection against reinfection with other homologous or heterologous species (31, 32). Multiple studies from Mexico, Perú, Ecuador, Bolivia, Brazil, and more recently Colombia have demonstrated the presence of mixed infection with different *Leishmania* species in CL patients (5, 6, 9, 10, 12). Such findings also raise questions regarding possible synergistic or antagonizing effects between species in the context of coinfections. Further research looking into potential synergistic interactions among species is of utmost importance in order to evaluate aspects such as virulence enhancement, immune response modulation, individual risk for infection, and differential response to antileishmanial therapy (33) in multispecies parasitism. Understanding the effects of polyparasitism will also undoubtedly improve our knowledge in the management of CL, as well as refine our diagnostic approach and determine the true prevalences of mixed infections, particularly in areas of endemicity.

Another interesting observation was uncovering the presence of coinfection in sand fly vectors and the close ecological interaction between *Leishmania* species, T. cruzi, and insect vectors (Fig. 3B). During the analysis, two interesting scenarios drew our attention. The first was the capacity of Pintomyia ovallesi to harbor two different *Leishmania* species. These findings coincide with previous reports depicting the intimate relationship of this vector with the parasite (34, 35), as well as the fundamental role that P. ovallesi plays in the transmission cycle of the parasite and progression across the clinical spectrum. Second, our results underscore the close relationship of Migonemyia migonei and Lutzomyia longipalpis with T. cruzi (Fig. 3B). Even though the presence of *Trypanosoma* in sand flies has not been described in Colombia, reports from other localities, including Italy (15, 36), Madrid (37), Thailand (14), and Brazil (38), have documented this association. Our findings suggest that sand flies may be acting as potential vectors of *Leishmania* and *Trypanosoma* parasites. However, the detection of T. cruzi DNA in seven of the sand flies analyzed is not sufficient evidence to incriminate these species as competent vectors, given that the genomic source could have originated from a blood meal, particularly in overlapping areas of endemicity where *Trypanosoma* and *Leishmania* species cocirculate, potentially causing mixed infections on both host and vectors. These results emphasize the need to prioritize vector competence studies of sand flies and *Trypanosoma* species.

The amplicon-based NGS analysis of Phyllostomus hastatus samples also revealed two interesting findings. The first was the identification of T. cruzi as the predominant parasite in this bat species (Fig. 3C), and the second was the presence of three bats coinfected with T. cruzi, L. braziliensis, and/or L. panamensis (Fig. 3C). Previous studies from Brazil (27, 39) and Colombia (40) have also recorded the presence of T. cruzi-infected Phyllostomus hastatus, as well as trypanosomatid polyparasitism in bats. So far, various studies have demonstrated mixed *Leishmania* species infections (L. braziliensis, L. amazonensis, L. chagasi, L. mexicana, and L. infantum) in bats, mainly from Brazil and Mexico (41–44), while other studies have recorded coinfection events involving *Trypanosoma* and *Leishmania* (45, 46). Still, no previous study has reported the presence of *Leishmania* DNA from bats in Colombia. To the best of our knowledge, this is the first study to demonstrate the presence of *Leishmania* and other trypanosomatids in wild reservoirs from areas of endemicity of Colombia using an amplicon-based-NGS methodology. From an ecological standpoint, we consider that T. cruzi and *Leishmania* coinfection in Phyllostomus hastatus may be linked to its feeding habits, given that this bat species is known to feed on wild mammals and a significant number of triatomines and other insects (sand flies) found in areas of endemicity of leishmaniasis and Chagas disease. In addition, it is important to highlight that these food sources occupy the same habitats (inside or around human dwellings) while sharing nighttime activity patterns with these bats (39, 41, 47), thus increasing the chances for consumption and infection. These findings demonstrate the advantages of using an amplicon-based NGS approach to detect mixed infections and provide further insights into the interaction of bats and different *Leishmania* species in order to design novel strategies for prevention and control of the disease.

When analyzing the results obtained from samples of domestic reservoirs (Canis familiaris), we observed the occurrence of coinfection events involving three different *Leishmania* species (L. panamensis/L. brazilienis/L. naiffi) (Fig. 3C). This was not surprising given that all these species have previously been associated with canine Leishmaniasis throughout different countries of South America (48–50). Despite the fact that canine leishmaniasis involving mixed species has been reported in both Brazil (51, 52) and Venezuela (11), to date, no cases of coinfection have been documented in Colombia. Therefore, this is the first report of *Leishmania* polyparasitism in naturally infected dogs from Colombia, showing triple-infection events. Interestingly, two of the canines showed coinfection with L. naiffi, a recently identified species in Colombia (53) that is known to cause human cutaneous disease in Panama, French Guiana, and the Brazilian Amazon (53, 54). Furthermore, the facts that L. naiffi had not been previously identified in Sincelejo, where the sample was obtained, and that one of its vectors (Lutzomyia gomezi) is endemic across neighboring departments (Cordoba, Atlantico, and La Guajira) (30, 55, 56) where cutaneous leishmaniasis is endemic strongly suggest a possible expansion of the ecological range of the sand fly vector, thus highlighting the introduction of new species and/or genotypes to certain geographical areas where they had not been previously reported, increasing the possibility that a vector/reservoir could be infected with more than one species. Identifying canines coinfected with different *Leishmania* species living in close proximity to humans has important public health implications for the design and implementation of epidemiological control programs of VL and CL in Colombia. In fact, assessing the true prevalence of coinfections is essential to improving surveillance and prevention strategies, particularly in at-risk populations from endemic rural and urban areas. Although we identified mixed infections in both vectors and reservoirs, we consider that a much deeper sampling with individuals from different geographic regions should be performed to confirm these findings. Finally, the diversity indices estimated from our results are revealing not only given the high diversity of species present in domestic reservoirs and sand flies (Fig. 5A) (57, 58) but also based on the fact that L. panamensis, L. braziliensis, and T. cruzi were the main species contributing to the dissimilarity between the groups (Fig. 5B). This was anticipated due to the wide geographic distribution of these species across the sampling areas, which signals an excellent power of adaptation of these species to different mammalian hosts and vectors. We consider that a deeper sampling of both sand fly vectors and domestic reservoirs is necessary to confirm these findings and determine the zoonotic contributions of trypanosomatids to clinical disease.

In summary, this study describes findings showing that HSP70 amplicon-based NGS, unlike Sanger sequencing, is a useful tool to detect mixed trypanosomatid infections. This is because amplicon-based NGS allows greater coverage of a specific region of interest, thus detecting new variants, rare species, or genotypes that are present in low abundance, as can occur in cases of mixed infection, and cannot be detected or unveiled by Sanger sequencing. Our findings showed this to be a reliable and efficient molecular method to screen for the presence and assess the genetic diversity of trypanosomatids in humans, mammalian (reservoir) hosts, and insect vectors. Our study showed a high level of concordance between our amplicon-based NGS method and Sanger sequencing, offering the additional advantage over Sanger sequencing of interrogating for mixed trypanosome infections, capturing lower-abundance genotypes, and identifying rare or previously unknown species commonly overlooked by Sanger sequencing. In addition, this assay could reduce costs and response times compared to broader approaches like whole-genome sequencing. In addition, the results presented herein depose the traditional paradigm of leishmaniasis as being a single-species-driven infection and redraw the choreography of host-pathogen interactions in the context of multiparasitism. Further research is needed to decipher how coinfections may influence disease progression. This knowledge is key to developing an integrated approach for diagnosis and treatment. Although the results obtained in this study describe the presence of mixed infections in the three groups analyzed (humans/vectors/reservoirs), it is also important to consider the possibility that some of the samples evaluated may contain hybrid strains, as previously described (59, 60), a hypothesis that would provide an additional advantage to our amplicon-based NGS method, since identifying hybrid strains circulating in certain geographic regions would not only change the epidemiological panorama of leishmaniasis but would also allow the techniques used for diagnosis and treatment of this pathology to be redesigned. Future studies should evaluate the feasibility of HSP70 to detect hybrid strains of *Leishmania*.

## MATERIALS AND METHODS

### Ethical statement.

This study was approved by the Ethics Committee of the Central Military Hospital of Colombia, following the principles established in the Declaration of Helsinki under Act No. 2043 of 22 March 2017. The procedures employed for collecting clinical material from reservoirs and sand fly vectors were carried out by the Universidad de Santander (UDES), which provided the collection permit from ANLA (Autoridad Nacional de Licencias Ambientales) (permit no. 01749).

### Sampling.

A total of 65 samples from humans, sand fly vectors, and reservoirs (wild and domestic) were included in the study. The samples included 49 samples obtained from direct smears of lesions from CL patients, 2 from Lara State (Venezuela) and 47 who attended the Dirección de Sanidad Militar, Ejercito Nacional de Colombia, Bogotá. As inclusion criteria, patients had to be male, over 18 years of age, to have had clinical and parasitological diagnosis of CL, with lesions of a minimum of 1 month and a maximum of 3 months of evolution, and to have been without antileishmanial treatment for at least 2 months prior to sampling. Only patients with a positive result for at least one direct smear or PCR of a skin biopsy specimen were included in the study. Those patients with lesions on the face, genitals, or mucosa and secondary infections of lesions were not sampled (61). Eight sand flies were collected across three different departments of Colombia (Norte de Santander, La Guajira, and Tolima). The collection, manipulation, and taxonomic identification of these specimens was performed according to previously reported methodology (34). Serum from two domestic reservoirs (Canis familiaris) with previous diagnosis of CL collected in the Sucre Department were also provided by the Laboratorio Nacional de Referencia del Instituto Nacional de Salud (INS), Colombia. Six blood spot samples from wild reservoirs (Phyllostomus hastatus) were collected on FTA cards in the Casanare Department. For blood collection, the animals were noninjuriously captured with a mist net and anesthetized with 20 mg/kg of body weight ketamine (Ketalar1; Parke-Davis, Morris Plains, NJ, USA). Approximately 0.3 ml of whole blood obtained by cardiac puncture was collected and placed on FTA cards. FTA cards were adequately preserved to maintain low humidity and avoid contamination. All metadata on the 65 samples included in the study is summarized in Table S1. The geographic localization of samples is represented in Fig. 4A. Finally, as control of the sequencing and with the aim of having an estimate of the limit of detection and reliability of the test, we made mixtures (1/1) of promastigotes of L. amazonensis and L. braziliensis at different concentrations, ranging from 1 × 10^10^ to 1 × 10^1^ cells/ml. For our analysis, we sequenced two concentrations: 1 × 10^6^ (mix-1) and 1 × 10^3^ (mix-2) were selected for further analysis.

### DNA extraction.

Once the samples were obtained, the next step was to extract the DNA from each of them. Two commercial kits were used. The ZR tissue/insect miniprep DNA Zymo kit (Zymo Research, Irvine, CA, USA) was used to extract the DNA from whole bodies of sand flies, and the High Pure PCR template preparation kit (Roche Life Science, Mannheim, Germany) was used to extract the DNA from samples collected from humans, reservoirs, and mixtures made for this study. Each kit was used following the protocol described by the manufacturer. Subsequently, the concentration, quality, and integrity of DNA obtained from each of the samples was determined. For this we used the Nanodrop ND-1000 spectrophotometer (Thermo Fisher Scientific, Inc., Waltham, MA, USA), which allowed us to measure the DNA concentration, and in addition, a 1% agarose gel electrophoresis was performed to verify the quality and integrity of products obtained. Finally, the DNA obtained was used for species identification by Sanger sequencing as reported elsewhere (62) and for amplicon-based NGS as developed herein.

### Specificity of the HSP70 primers employed in the assay.

In order to determine the intra- and interspecies specificity of the primers used in this study, we downloaded the annotation of the main *Leishmania* and *Trypanosoma* species from tritrypdb, a publicly available database (https://tritrypdb.org/common/downloads/Current_Release), selecting by species only those sequences corresponding to the HSP70 gene. Each of these sequences per chromosome, per species was aligned to the primers under study. Those sequences recognized by the primers were used to build a maximum-likelihood-based phylogenetic tree using FastTree double precision version 2.1.10 with 1,000 bootstrap replicates (63). The final tree obtained was visualized using the interactive tool Interactive Tree Of Life v4 (http://itol.embl.de) (64).

### Identification of *Leishmania* species by Sanger sequencing.

We carried out direct Sanger sequencing to identify the trypanosomatid species associated with infection in CL patients, sand fly vectors, and reservoirs. For this, the gene encoding heat shock protein (HSP70) was amplified. HSP70F (5′ AGG TGA AGG CGA CGA ACG 3′) and HSP70R (5′ CGC TTG TCC ATC TTT GCG TC 3′) primers were used to amplify a 337- bp region of the HSP70 gene (65). The thermal profile was as follows: denaturation at 94°C for 5 min, followed by 40 cycles with denaturation at 94°C for 1 min, annealing at 58°C for 1 min, and elongation at 72°C for 1 min and a final extension at 72°C for 10 min (29, 65). EXOSAP (Affymetrix, Santa Clara, CA, USA), was used to purify the amplified products, and the sequencing was performed using the dideoxy-terminal method (AB3730; Applied Biosystem, Foster City, CA, USA). The sequences obtained were submitted to BLASTn (using the webserver) for a similarity search against trypanosomatid sequences deposited in GenBank (29). The best matches of E value and percentage of identity were selected, thus allowing the assignment of species to each of the analyzed samples. Once a trypanosomatid species was identified, the results were compared with those obtained using the amplicon-based NGS.

### Amplicon-based next-generation sequencing.

Genomic DNA (≥200 ng/μl) from CL patients, sand fly vectors, and reservoirs (wild and domestic) was used for sequencing, which was conducted by Novogen (Beijing, China). Briefly, primers F (5′ AGGTGAAGGCGACGAACG) and R (5′ CGCTTGTCCATCTTTGCGTC) were used to amplify the HSP70 gene using the Phusion high-fidelity PCR master mix (New England Biolabs). The quality, integrity, and size of the amplicon (337 bp) were determined by 2% agarose gel electrophoresis. The amplicons from each sample were purified using the Qiagen gel extraction kit (Qiagen, Germany) according to the manufacturer’s protocol. Later, the sequencing libraries were generated using the NEBNext ultra DNA library prep kit for Illumina, following the manufacturer’s recommendations, and index codes were added (Table S2). The libraries were purified using Agencourt AMPure XP beads (to remove excess primers, nucleotides, salts, and enzymes produced during the reactions), quantified using the Qubit 2.0 fluorometer (Thermo Scientific), and qualified using the Agilent Bioanalyzer 2100 system. Finally, the library was sequenced on an Illumina platform, and 250-bp paired-end reads were generated following the manufacturer’s instructions.

### Bioinformatics analysis.

FASTQ files (forward and reverse) for every sample were obtained from Illumina sequencing of HSP70 amplicons. The sequences were initially filtered for quality using the QIIME software (66), following the quality parameters described previously (22), and subsequently merged. Those sequences that passed the quality filtering were compared with an in-house-built database (see “Reference data set” below) for trypanosomatid species detection.

The taxonomic assignation of high-quality reads was conducted using BLASTn locally against the built database, considering as the threshold a minimum 95% identity and an E value of 10. Those matches that had a relative abundance (number of reads assigned per sample divided by the total amount of reads of the sample) greater than 3% were considered in the analysis in order to rule out potential sequencing errors; additionally, we adjusted the relative abundance of reads of the *Leishmania* species identified to the number of copies of the HSP70 gene present in each of them to have a better approximation of the abundance of the species per individual. All quantitative results were graphed using R software version 3.6.1.

### Reference data set.

An in-house database was built considering all the HSP70 sequences of trypanosomatids in the NCBI Nucleotide database (https://www.ncbi.nlm.nih.gov/nucleotide/). We conducted a search considering two criteria: the gene name “Heat shock protein 70” and the organism name “Kinetoplastids,” obtaining a total of 990 sequences. Later, the search was filtered considering the molecule type (genomic DNA/RNA), the source database (GenBank), and the sequence length (330 to 5,000). Additionally, we included six HSP70 sequences from reference genomes, including those of L. braziliensis MHOM/BR/75/M2904 (XM_001566275), L. major strain Friedlin (XM_001684512), L. infantum JPCM5 (XM_003392631), L. mexicana MHOM/GT/2001/U1103 (XM_003877073.1), L. donovani LDBPK_283000 (XM_003862348.1), and T. cruzi strain CL Brener (XM_812645), which were downloaded from the Reference Sequence (RefSeq) database. We excluded those sequences of *Leishmania* that did not have a correct taxonomic assignment to species level (*Leishmania* sp.). The sequences obtained were aligned in Clustal W and trimmed according to the HSP70 forward/reverse primer sequence in UGENE version 33.0 software (67). Sequences with high levels of gaps in content and low quality were excluded. A total of 385 sequences constituted our final reference database, which is publicly available at https://github.com/gimur/Amplicon-Base-Next-Generation-Sequencing-.git. To confirm the utility of HSP70 for identifying trypanosomatid species, we recovered sequences by haplotype from the 385 sequences obtained, using the DNAsp software version 5.0, obtaining a total of 48 haplotypes. Considering the total number of single-nucleotide polymorphisms (SNPs) between the haplotypes identified and using the snp-dists SNP distance program (https://github.com/tseemann/snp-dists), we generated a distance matrix. Additionally, we built two maximum-likelihood-based phylogenetic trees using FastTree double precision version 2.1.10 (63). One of them was constructed to explore the clustering among the 385 sequences used as a reference database and the 113 sequences obtained from 65 samples included in this study, and the second was performed to observe the clustering among the 48 haplotypes from 385 sequences used as the reference database. The robustness of the nodes was evaluated using the bootstrap test (BT, with 1,000 replicates). The tree obtained was visualized using the interactive tool Interactive Tree Of Life v4 (http://itol.embl.de) (64). Finally, phylogenetic networks were also built in SplitsTree5 (68) using the Neighbor-Net method.

### Statistical analysis.

A descriptive analysis of the clinical and epidemiological variables was performed. For continuous values, the distribution of the data and the hypothesis of normality were initially evaluated using the Shapiro-Wilk test. Because the data did not have a normal distribution, continuous variables were summarized in terms of medians and interquartile ranges (IQRs). Qualitative variables were summarized in frequencies and proportions according to the infectious species and single- or coinfection patterns. Due to the distribution of the quantitative data, to identify the statistical significance of possible differences between the single-infection and coinfection groups, the nonparametric Mann-Whitney-Wilcoxon test was applied (69).

Additionally, to compare infection species corresponding to several groups, we applied the Kruskal-Wallis test and the corresponding test for pairwise multiple comparisons between groups using the Dunn-Bonferroni *post hoc* method. The chi-square test and Fisher exact test were performed to identify potential associations between the categorical variables and species groups. R software (RStudio Team 2019) was used to perform the statistical analysis. A *P* value of <0.05 was considered statistically significant. Finally, we used STATA 11 software to calculate the agreement between the two methods analyzed here (Sanger sequencing and HSP70 amplicon-based NGS), considering the agreement percentages and the Kappa coefficients (κ). A 0.05 significance level was fixed (70).

### Diversity analysis.

The diversity analyses were done using the R package phyloseq (71) version 4.0.2. The alpha diversity of amplicon sequence variant (ASVs) of each group analyzed (humans, sand flies, and wild and domestic reservoirs) was calculated using Simpson dominance and Shannon diversity. The Kolmogorov-Smirnov test was implemented to verify the normality of data obtained from the diversity analysis. The nonparametric Kruskal-Wallis test compared the alpha diversity metrics for the four groups. Finally, we conducted multiple pairwise comparisons of alpha diversity between groups using the Dunn-Bonferroni *post hoc* method, to determine the groups among which these differences were present. A *P* value of <0.05 was considered statistically significant.

Likewise, using the phyloseq package in R, principal coordinates analysis (PCoA) and principal-component analysis (PCA) diagrams were produced, in which Bray-Curtis distances were used between the samples to visualize the behavior of the groups. We performed permutational multivariate analysis of variance (PERMANOVA) to identify differences in the distribution and dispersion of the groups. Additionally, similarity percentage (SIMPER) analysis was implemented to establish which species were likely to be the main contributors to any differences between groups detected by PERMANOVA.
