# Supplementary material for: Development of an Amplicon-Based Next-Generation Sequencing Protocol to Identify Leishmania Species and Other Trypanosomatids in Leishmaniasis Endemic Areas
Source: Microbiol Spectr. 2021 Oct 13;9(2):e00652-21. doi: 10.1128/Spectrum.00652-21 (PMC8515931; doi:10.1128/Spectrum.00652-21)
Supplement: Supplemental file 1 — Supplemental material. Download SPECTRUM00652-21_Supp_1_seq9.pdf, PDF file, 0.5 MB [file spectrum00652-21_supp_1_seq9.pdf]

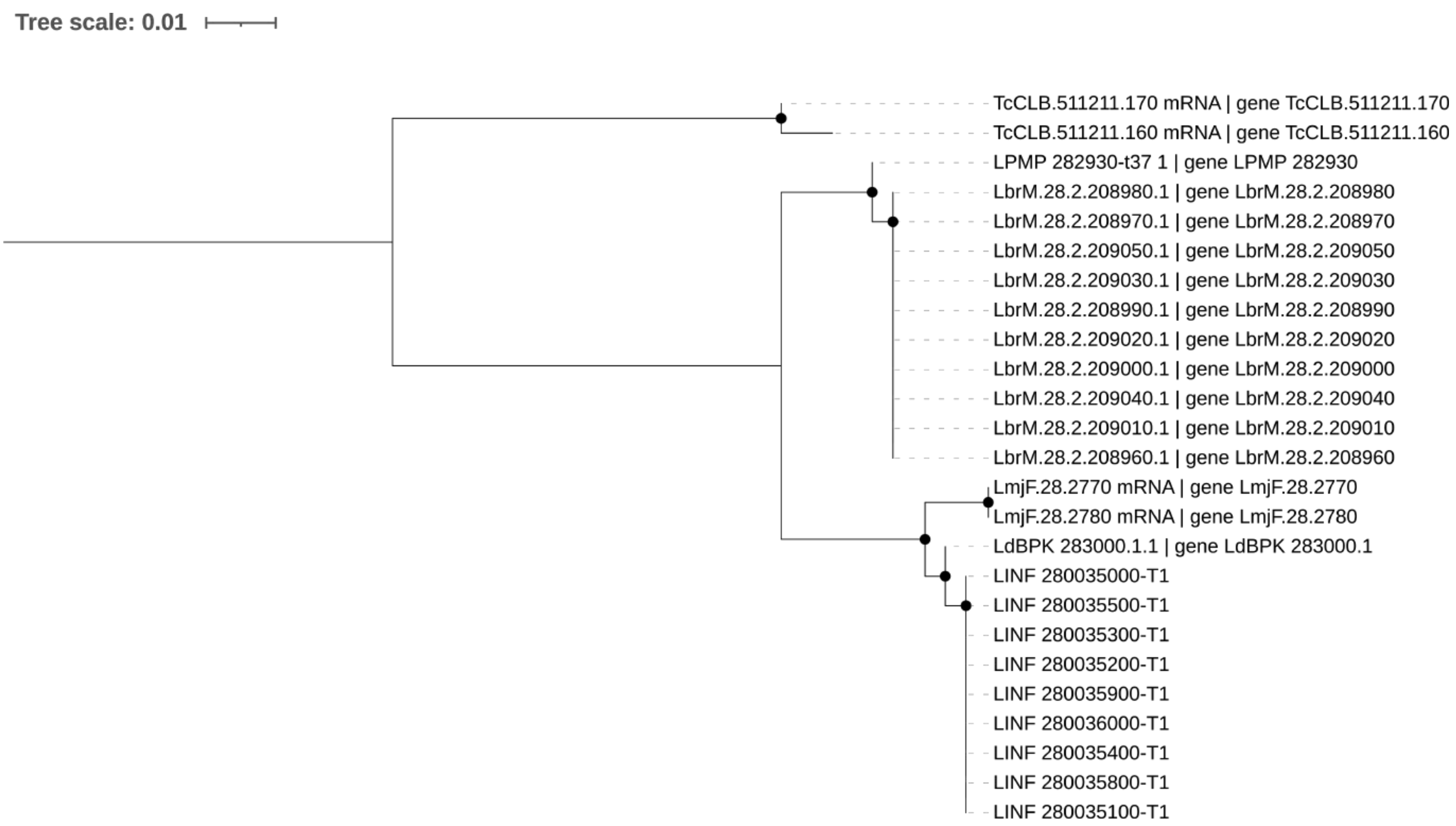

Figure supplementary S1. Phylogenetic relationship between the different HSP70 gen copies in trypanosomatids. The figure represents the phylogenetic analysis of HSP70 sequences in trypanosomatids, recognized by the primers used in the study. The black dots represent well-supported nodes (Bootstrap ≥ 90).

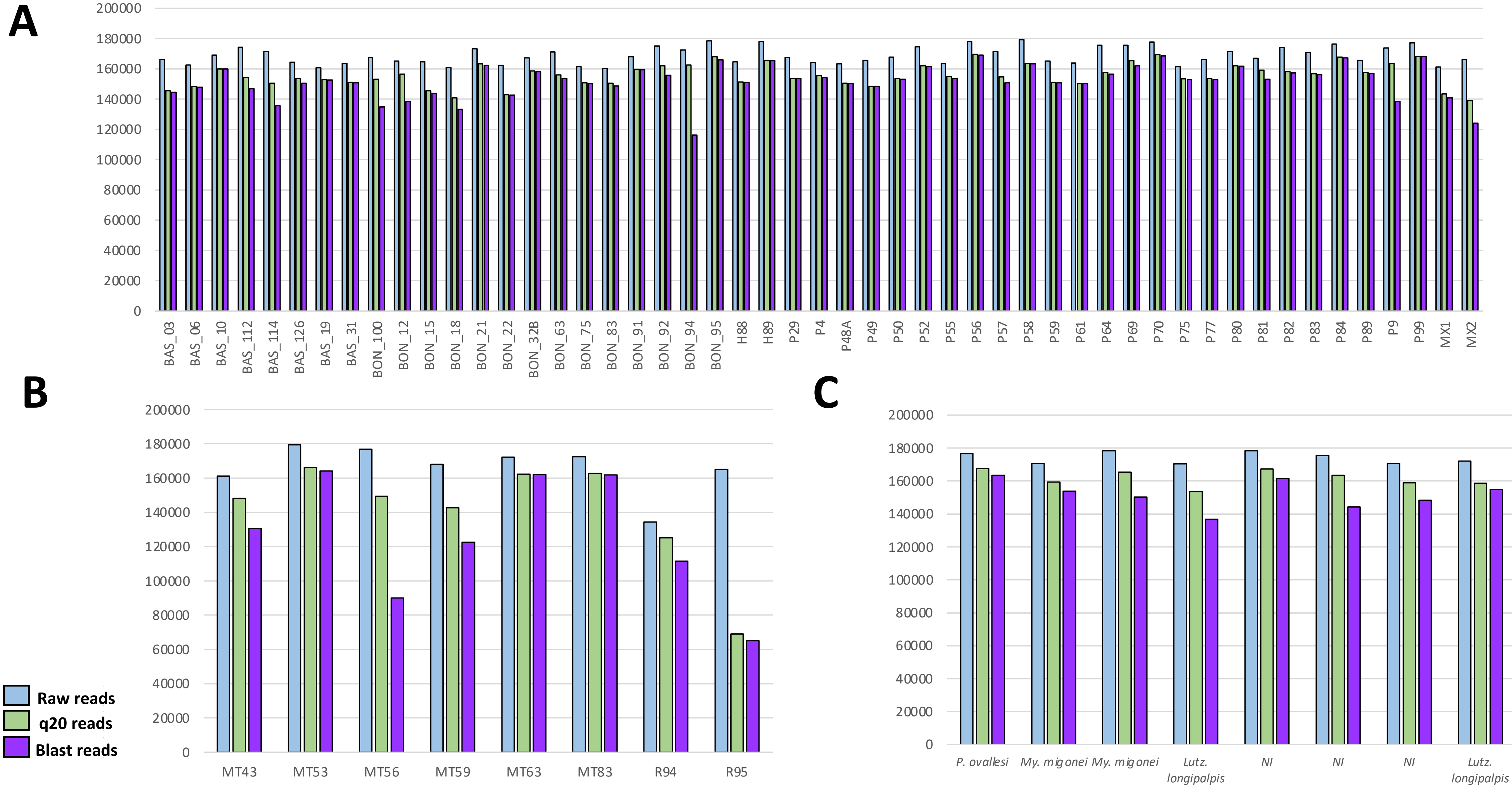

Figure supplementary S2. Statistical analysis of Amplicon-base NGS for each sample included in the study. The blue bars represent the total number of reads obtained during the sequencing, the green bars the number of reads that passed the quality filter (minimal average quality score of 20), and the purple bars the number of reads that were taxonomic assignation using BLASTn, in samples from CL Patients (A), sandfly vectors (B) and reservoirs (C).

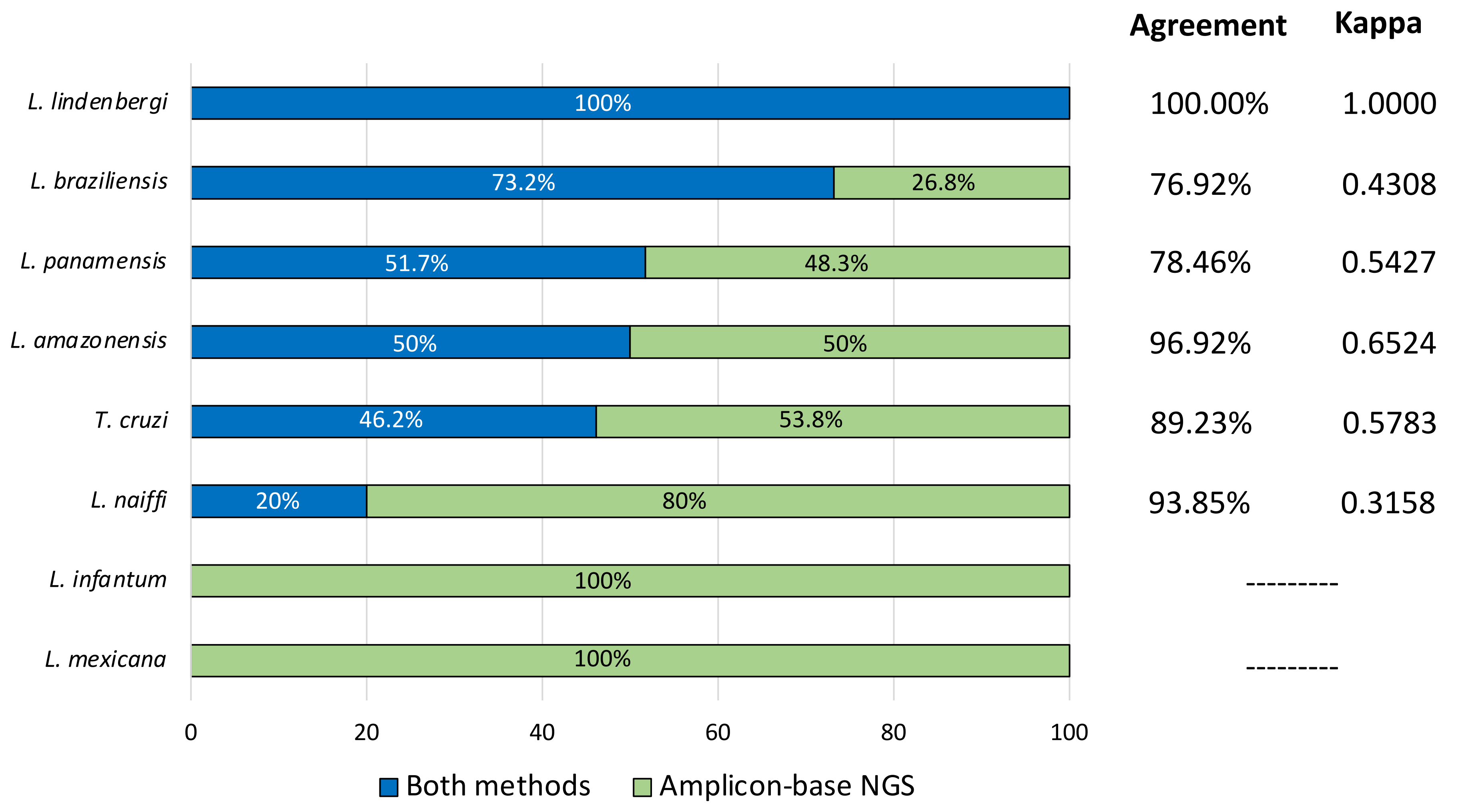

Figure supplementary S3. Agreement between Sanger sequencing and HSP70 amplicon-based NGS. The figure represents the agreement results expressed in percentages between both methods and HSP70 amplicon-based NGS, accompanied by their corresponding Kappa coefficient.

Table S1: Metadata on 65 samples included in the study.

CL Patients										
							Medical resolution	Possible site of infection		
Sample code	Sampling date	Patient Age	Number of lesions	anatomical location of the lesions	Shape of lesion	Disease evolution (months)	Therapeutic failure (0) Healing (1)	Municipality	Department	Treatment
BAS 03	19/07/2017	26	13	right arm	Ulcer	3	1	Campo alegre	Guainía	Meglumine antimoniate
BAS 06	19/07/2017	21	9	right forearm	Ulcer	3	1	Campo alegre	Guainía	Meglumine antimoniate
BAS 10	25/07/2017	19	7	right hand	Ulcer	3	0	Campo alegre	Guainía	Meglumine antimoniate
BAS 19	03/08/2017	21	1	right leg	Ulcer	3	0	Miraflores	Guaviare	Meglumine antimoniate
BAS 31	15/11/2017	20	1	right hand	Ulcer	10	0	Cimitarra	Santander	Pentamidine isethionate
BAS 112	12/02/2019	24	2	neck	Ulcer	4	1	San José del Guaviare	Guaviare	Pentamidine isethionate
BAS 114	12/02/2019	26	5	right arm	Warty	WD	1	San José del Guaviare	Guaviare	Pentamidine isethionate
BAS 126	12/02/2019	24	1	left forearm	Ulcer	WD	0	San José del Guaviare	Guaviare	Pentamidine isethionate
BON 12	04/08/2017	23	1	scalp	Ulcer	1	1	Puerto Rico	Meta	Meglumine antimoniate
BON 15	04/08/2017	25	2	scalp	Ulcer	1	0	La Macarena	Meta	Meglumine antimoniate
BON 18	04/08/2017	26	1	left arm	Ulcer	0.66	1	La Macarena	Meta	Meglumine antimoniate
BON 21	04/08/2017	23	2	right leg	Ulcer	0.5	1	Yarumales	Meta	Meglumine antimoniate
BON 22	04/08/2017	25	1	back	Ulcer	1.5	0	Puerto Leguizamo	Putumayo	Meglumine antimoniate
BON 32	15/08/2017	19	2	left hand	Ulcer	2.5	1	Miraflores	Guaviare	Meglumine antimoniate
BON 63	22/09/2017	19	1	chest	Ulcer	WD	1	San José del Guaviare	Guaviare	Meglumine antimoniate
BON 75	03/10/2017	31	2	left leg	Ulcer	WD	1	La Julia	Meta	Meglumine antimoniate
BON 83	18/10/2017	25	2	left arm	Ulcer	1	1	Carrizales	Antioquia	Meglumine antimoniate
BON91	30/10/2017	22	1	left leg	Ulcer	1.5	WD	Nóvita	Chocó	Meglumine antimoniate
BON92	10/11/2017	25	1	left forearm	Ulcer	2	1	Tumaco	Nariño	Meglumine antimoniate
BON94	10/11/2017	19	1	abdomen	Ulcer	3	WD	Patía	Cauca	Meglumine antimoniate
BON95	10/11/2017	27	1	right hand	Ulcer	1	WD	Macarena	Meta	Meglumine antimoniate
BON 100	27/11/2017	25	1	right leg	Ulcer	2	1	San José del Guaviare	Guaviare	Meglumine antimoniate
P4	02/05/2018	39	1	neck	Ulcer	1.5	0	Tibú	Norte de Santander	Meglumine antimoniate
P9	02/05/2018	25	1	arm	Ulcer	1	1	Tumaco	Nariño	Meglumine antimoniate
P29	06/06/2018	22	1	head	Ulcer	2	1	Riohacha	Guajira	Meglumine antimoniate
P48A	06/06/2018	37	1	NI	Ulcer	NI	NI	Araudia	Caquetá	Meglumine antimoniate
P49	09/08/2018	23	1	shoulder	Ulcer	NI	1	San José	Guaviare	Meglumine antimoniate
P50	09/08/2018	22	2	head	Ulcer	NI	1	San José	Guaviare	Meglumine antimoniate
P52	09/08/2018	20	1	NI	Ulcer	NI	1	San José	Guaviare	Meglumine antimoniate
P55	09/08/2018	22	1	hand	Ulcer	NI	0	Sabanalarga	Atlantico	Meglumine antimoniate
P56	09/08/2018	22	2	hand	Ulcer	NI	1	San José	Guaviare	Meglumine antimoniate
P57	09/08/2018	23	2	hand	Ulcer	1	1	San José	Guaviare	Meglumine antimoniate
P58	09/08/2018	22	1	hand	Ulcer	NI	1	San José	Guaviare	Meglumine antimoniate
P59	09/08/2018	21	1	forearm	Ulcer	NI	0	San José	Guaviare	Meglumine antimoniate
P61	09/08/2018	25	1	forearm	Ulcer	1	1	La macarena	Meta	Meglumine antimoniate
P64	09/08/2018	21	1	back	Ulcer	NI	NI	San José	Guaviare	Meglumine antimoniate
P69	04/09/2018	24	1	left ear	Ulcer	3	NI	Cucúta	Norte de Santander	Meglumine antimoniate
P70	04/09/2018	22	1	left right	Ulcer	NI	NI	Tumaco	Nariño	Meglumine antimoniate
P75	04/09/2018	26	1	hand	Ulcer	NI	1	Nariño	Nariño	Meglumine antimoniate
P77	04/09/2018	24	1	hand	Ulcer	NI	1	Buena Vista	Guajira	Meglumine antimoniate
P80	04/09/2018	22	1	head	Ulcer	NI	1	San José	Guaviare	Meglumine antimoniate
P81	04/09/2018	25	1	hand	Ulcer	NI	1	San José	Guaviare	Meglumine antimoniate
P82	04/09/2018	20	2	hand	Ulcer	NI	1	Guajira	Guajira	Meglumine antimoniate
P83	04/09/2018	21	1	hand	Ulcer	NI	1	Ipiales	Nariño	Meglumine antimoniate
P84	04/09/2018	27	1	hand	Ulcer	NI	1	Tolemaida	Cundinamarca	Meglumine antimoniate
P89	05/10/2018	24	1	neck	Ulcer	2.5	1	San José	Guaviare	Meglumine antimoniate
P99	12/10/2018	27	2	NI	Ulcer	1.6	0	NI	NI	Meglumine antimoniate
H88	17/10/2017	8	1	hand	Ulcer	7	NI	Iribarren	Lara (Venezuela)	No treatment
H89	24/10/2017	2	1	hand	Ulcer	4	1	Iribarren	Lara (Venezuela)	Meglumine antimoniate

NI= No information

Sand fly vectors						
Sample ID	Asociation	Sex	Sampling place	Department	Municipality	Sample origen
L75	Lutzomyia	Female	NI	Norte de Santander	Siravita	*Pintomyia (Pifanomyia) ovallesi*
V58	Lutzomyia	Male	NI	La Guajira	La Guajira	*My. migonei*
V59	Lutzomyia	Male	NI	La Guajira	La Guajira	*My. migonei*
V60	Lutzomyia	Female	Peridomicile	La Guajira	Maicao	*Lutzomyia longipalpis*
V61	Lutzomyia	Female	Peridomicile	La Guajira	Maicao	NI
V62	Lutzomyia	Female	Peridomicile	La Guajira	Maicao	NI
V63	Lutzomyia	Female	Intradomicile	La Guajira	Maicao	NI
V64	Lutzomyia	Female	NI	Tolima	Tolemaida	*Lutzomyia longipalpis*

NI= No information

Reservoirs							
Sample ID	Asociation	Sex	Patient age	Department	Municipality	Sampling place	Sample origen
MT43	Wild Reservoir	Male	Adult	Casanare	La niata	NI	*Phyllostomus hastatus*
MT53	Wild Reservoir	Male	Adult	Casanare	La niata	NI	*Phyllostomus hastatus*
MT56	Wild Reservoir	Male	Adult	Casanare	La niata	NI	*Phyllostomus hastatus*
MT59	Wild Reservoir	Male	Adult	Casanare	La niata	NI	*Phyllostomus hastatus*
MT63	Wild Reservoir	Female	Adult	Casanare	San isidro	NI	*Phyllostomus hastatus*
MT83	Wild Reservoir	Male	Adult	Casanare	Curama	NI	*Phyllostomus hastatus*
R94	Domestic Reservoir	Female	2 years	Sincelejo	Sucre	Intradomicile	*Canis familiaris*
R95	Domestic Reservoir	Male	8 month	Sincelejo	Sucre	Intradomicile	*Canis familiaris*

NI= No information

Table S2: List of barcodes and primer sequence, used in the Amplicon-based NGS.

	#sampleID	Barcode Sequence	Primer Sequence
CL Patients	BAS 03	AGTGGTCA,CTAAGGTC	AGGTGAAGGCGACGAACG,CGCTTGTCCATCTTTGCGTC
	BAS 06	ACATTGGC,CTAAGGTC	AGGTGAAGGCGACGAACG,CGCTTGTCCATCTTTGCGTC
	BAS 10	ATGCCTAA,CGACACAC	AGGTGAAGGCGACGAACG,CGCTTGTCCATCTTTGCGTC
	BAS 112	AAACATCG,CTAAGGTC	AGGTGAAGGCGACGAACG,CGCTTGTCCATCTTTGCGTC
	BAS 114	AAACATCG,CGGATTGC	AGGTGAAGGCGACGAACG,CGCTTGTCCATCTTTGCGTC
	BAS 126	AGTGGTCA,CGACACAC	AGGTGAAGGCGACGAACG,CGCTTGTCCATCTTTGCGTC
	BAS 19	ACAAGCTA,CCTCTATC	AGGTGAAGGCGACGAACG,CGCTTGTCCATCTTTGCGTC
	BAS 31	CTGTAGCC,CCTAATCC	AGGTGAAGGCGACGAACG,CGCTTGTCCATCTTTGCGTC
	BON 100	ACAAGCTA,CGACACAC	AGGTGAAGGCGACGAACG,CGCTTGTCCATCTTTGCGTC
	BON 12	CTGTAGCC,CGGATTGC	AGGTGAAGGCGACGAACG,CGCTTGTCCATCTTTGCGTC
	BON 15	ACCACTGT,CTAAGGTC	AGGTGAAGGCGACGAACG,CGCTTGTCCATCTTTGCGTC
	BON 18	AACGTGAT,CTAAGGTC	AGGTGAAGGCGACGAACG,CGCTTGTCCATCTTTGCGTC
	BON 21	ACAAGCTA,CGACACAC	AGGTGAAGGCGACGAACG,CGCTTGTCCATCTTTGCGTC
	BON 22	AGTGGTCA,CGGATTGC	AGGTGAAGGCGACGAACG,CGCTTGTCCATCTTTGCGTC
	BON 32B	AACGTGAT,CCTCTATC	AGGTGAAGGCGACGAACG,CGCTTGTCCATCTTTGCGTC
	BON 63	ACCACTGT,CGGATTGC	AGGTGAAGGCGACGAACG,CGCTTGTCCATCTTTGCGTC
	BON 83	CGCTGATC,CGACACAC	AGGTGAAGGCGACGAACG,CGCTTGTCCATCTTTGCGTC
	BON 91	ACATTGGC,CCTCTATC	AGGTGAAGGCGACGAACG,CGCTTGTCCATCTTTGCGTC
	BON 92	CATCAAGT,CGGATTGC	AGGTGAAGGCGACGAACG,CGCTTGTCCATCTTTGCGTC
	BON 94	ACAAGCTA,CCTAATCC	AGGTGAAGGCGACGAACG,CGCTTGTCCATCTTTGCGTC
	BON 95	AAACATCG,CCTCTATC	AGGTGAAGGCGACGAACG,CGCTTGTCCATCTTTGCGTC
	BON75	ATGCCTAA,GAGTTAGC	AGGTGAAGGCGACGAACG,CGCTTGTCCATCTTTGCGTC
	H88	ACAAGCTA,GAGTTAGC	AGGTGAAGGCGACGAACG,CGCTTGTCCATCTTTGCGTC
	H89	CTGTAGCC,GAGTTAGC	AGGTGAAGGCGACGAACG,CGCTTGTCCATCTTTGCGTC
	P29	CTGTAGCC,CCTCTATC	AGGTGAAGGCGACGAACG,CGCTTGTCCATCTTTGCGTC
	P4	ACAAGCTA,CGGATTGC	AGGTGAAGGCGACGAACG,CGCTTGTCCATCTTTGCGTC
	P48A	AAACATCG,CGACACAC	AGGTGAAGGCGACGAACG,CGCTTGTCCATCTTTGCGTC
	P49	ATGCCTAA,CTAAGGTC	AGGTGAAGGCGACGAACG,CGCTTGTCCATCTTTGCGTC
	P50	CGCTGATC,CGGATTGC	AGGTGAAGGCGACGAACG,CGCTTGTCCATCTTTGCGTC
	P52	AAACATCG,CGACACAC	AGGTGAAGGCGACGAACG,CGCTTGTCCATCTTTGCGTC
	P55	ACATTGGC,CGACACAC	AGGTGAAGGCGACGAACG,CGCTTGTCCATCTTTGCGTC
	P56	ACCACTGT,CCTCTATC	AGGTGAAGGCGACGAACG,CGCTTGTCCATCTTTGCGTC
	P57	ACAAGCTA,CTAAGGTC	AGGTGAAGGCGACGAACG,CGCTTGTCCATCTTTGCGTC
	P58	CTGTAGCC,CTAAGGTC	AGGTGAAGGCGACGAACG,CGCTTGTCCATCTTTGCGTC
	P59	CTGTAGCC,CGACACAC	AGGTGAAGGCGACGAACG,CGCTTGTCCATCTTTGCGTC
	P61	ACAAGCTA,CCTCTATC	AGGTGAAGGCGACGAACG,CGCTTGTCCATCTTTGCGTC
	P64	ACAAGCTA,CGGATTGC	AGGTGAAGGCGACGAACG,CGCTTGTCCATCTTTGCGTC
	P69	AGTGGTCA,CCTCTATC	AGGTGAAGGCGACGAACG,CGCTTGTCCATCTTTGCGTC
	P70	CTGTAGCC,CCTCTATC	AGGTGAAGGCGACGAACG,CGCTTGTCCATCTTTGCGTC
	P75	CATCAAGT,CCTCTATC	AGGTGAAGGCGACGAACG,CGCTTGTCCATCTTTGCGTC
	P77	AGTGGTCA,GAGTTAGC	AGGTGAAGGCGACGAACG,CGCTTGTCCATCTTTGCGTC
	P80	AACGTGAT,CGACACAC	AGGTGAAGGCGACGAACG,CGCTTGTCCATCTTTGCGTC
	P81	ACAAGCTA,CTAAGGTC	AGGTGAAGGCGACGAACG,CGCTTGTCCATCTTTGCGTC
	P82	CTGTAGCC,CGGATTGC	AGGTGAAGGCGACGAACG,CGCTTGTCCATCTTTGCGTC
	P83	CATCAAGT,CTAAGGTC	AGGTGAAGGCGACGAACG,CGCTTGTCCATCTTTGCGTC
	P84	CTGTAGCC,CGACACAC	AGGTGAAGGCGACGAACG,CGCTTGTCCATCTTTGCGTC
	P89	CTGTAGCC,CTAAGGTC	AGGTGAAGGCGACGAACG,CGCTTGTCCATCTTTGCGTC
	P9	AACGTGAT,CGACACAC	AGGTGAAGGCGACGAACG,CGCTTGTCCATCTTTGCGTC
	P99	ATGCCTAA,CCTCTATC	AGGTGAAGGCGACGAACG,CGCTTGTCCATCTTTGCGTC
Sand fly vectors	V58	ATGCCTAA,GAACAGGC	AGGTGAAGGCGACGAACG,CGCTTGTCCATCTTTGCGTC
	V59	AGTGGTCA,GAACAGGC	AGGTGAAGGCGACGAACG,CGCTTGTCCATCTTTGCGTC
	V60	ACCACTGT,GAACAGGC	AGGTGAAGGCGACGAACG,CGCTTGTCCATCTTTGCGTC
	V61	ACATTGGC,GAACAGGC	AGGTGAAGGCGACGAACG,CGCTTGTCCATCTTTGCGTC
	V62	CATCAAGT,GAACAGGC	AGGTGAAGGCGACGAACG,CGCTTGTCCATCTTTGCGTC
	V63	CGCTGATC,GAACAGGC	AGGTGAAGGCGACGAACG,CGCTTGTCCATCTTTGCGTC
	V64	ACAAGCTA,GAACAGGC	AGGTGAAGGCGACGAACG,CGCTTGTCCATCTTTGCGTC
	L75	AACGTGAT,CCTAATCC	AGGTGAAGGCGACGAACG,CGCTTGTCCATCTTTGCGTC
Reservoirs	MT43	AGTGGTCA,CCTAATCC	AGGTGAAGGCGACGAACG,CGCTTGTCCATCTTTGCGTC
	MT53	ACCACTGT,CCTAATCC	AGGTGAAGGCGACGAACG,CGCTTGTCCATCTTTGCGTC
	MT56	ACATTGGC,CCTAATCC	AGGTGAAGGCGACGAACG,CGCTTGTCCATCTTTGCGTC
	MT59	AACGTGAT,GAGTTAGC	AGGTGAAGGCGACGAACG,CGCTTGTCCATCTTTGCGTC
	MT63	CATCAAGT,CCTAATCC	AGGTGAAGGCGACGAACG,CGCTTGTCCATCTTTGCGTC
	MT83	AAACATCG,GAGTTAGC	AGGTGAAGGCGACGAACG,CGCTTGTCCATCTTTGCGTC
	R94	AGTGGTCA,CCTAATCC	AGGTGAAGGCGACGAACG,CGCTTGTCCATCTTTGCGTC
	R95	ACCACTGT,CCTAATCC	AGGTGAAGGCGACGAACG,CGCTTGTCCATCTTTGCGTC
Mix	Mx1	AACGTGAT,GAACAGGC	AGGTGAAGGCGACGAACG,CGCTTGTCCATCTTTGCGTC
	Mx2	AAACATCG,GAACAGGC	AGGTGAAGGCGACGAACG,CGCTTGTCCATCTTTGCGTC

Table S3: Comparison of the results obtained between sanger sequencing and Amplicon-base NGS, in each sample included in the study.

ID Genome	*Leishmania* species	
	**Sanger sequencing**	**Amplicon-base NGS**
**BAS 03**	*L. braziliensis*	*L.braziliensis*
**BAS 06**	*L. braziliensis*	*L.braziliensis*
**BAS 10**	*L. braziliensis*	*L.braziliensis*
**BAS 19**	*L. naiffi*	*L.naiffi/L.panamensis/L.braziliensis*
**BAS 31**	*L. panamensis*	*L.panamensis*
**BAS 112**	*L. braziliensis*	*L.braziliensis*
**BAS 114**	*L. braziliensis*	*L.braziliensis*
**BAS 126**	*L. braziliensis*	*L.braziliensis*
**BON 12**	*L. braziliensis*	*L.braziliensis*
**BON 15**	*L. braziliensis*	*L.braziliensis*
**BON18**	*L. braziliensis*	*L.braziliensis*
**BON21**	*L. braziliensis*	*L.braziliensis/L.panamensis*
**BON22**	*L. braziliensis*	*L.braziliensis*
**BON32B**	*L. lindenbergi*	*L.lindenbergi/L.braziliensis/L.amazonensis*
**BON63**	*L. braziliensis*	*L.braziliensis*
**BON75**	*L. braziliensis*	*L.braziliensis*
**BON83**	*L. panamensis*	*L.panamensis*
**BON91**	*L. panamensis*	*L.panamensis*
**BON92**	*L. panamensis*	*L.panamensis*
**BON94**	*L. panamensis*	*L.panamensis*
**BON95**	*L. braziliensis*	*L.braziliensis*
**BON100**	*L. braziliensis*	*L.braziliensis*
**H88**	*L. amazonensis*	*L.mexicana/L.amazonensis/L.infantum/L.braziliensis*
**H89**	*L. amazonensis*	*L.mexicana/L.amazonensis/L.infantum/L.braziliensis*
**P4**	*L. braziliensis*	*L.braziliensis*
**P9**	*L. braziliensis*	*L.braziliensis/L.panamensis/L.infantum*
**P29**	*L. panamensis*	*L.panamensis/L.braziliensis*
**P48A**	*L. braziliensis*	*L.braziliensis*
**P49**	*L. braziliensis*	*L.braziliensis/L.panamensis*
**P50**	*L. braziliensis*	*L.braziliensis*
**P52**	*L. braziliensis*	*L.braziliensis*
**P55**	*L. panamensis*	*L.panamensis*
**P56**	*L. braziliensis*	*L.braziliensis/L.naiffi*
**P57**	*L. braziliensis*	*L.braziliensis*
**P58**	*L. braziliensis*	*L.braziliensis/L.panamensis*
**P59**	*L. braziliensis*	*L.braziliensis*
**P61**	*L. braziliensis*	*L.braziliensis/L.panamensis*
**P64**	*L. braziliensis*	*L.braziliensis/L.panamensis*
**P69**	*L. braziliensis*	*L.braziliensis*
**P70**	*L. panamensis*	*L.panamensis/L.braziliensis*
**P75**	*L. panamensis*	*L.panamensis*
**P77**	*L. panamensis*	*L.panamensis/L.braziliensis*
**P80**	*L. braziliensis*	*L.braziliensis*
**P81**	*L. braziliensis*	*L.braziliensis/L.panamensis/L.naiffi*
**P82**	*L. panamensis*	*L.panamensis/L.braziliensis*
**P83**	*L. panamensis*	*L.panamensis*
**P84**	*L. panamensis*	*L.panamensis/L.braziliensis*
**P89**	*L. braziliensis*	*L.braziliensis*
**P99**	*L. panamensis*	*L.panamensis*
**L75**	*L. braziliensis*	*L. braziliensis/L. panamensis/ L.infantum*

**V58**	*L. braziliensis*	*L. braziliensis/T.Cruzi*
**V59**	*L. braziliensis*	*L. braziliensis/T.Cruzi/L. amazonensis*
**V60**	*L. braziliensis*	*L. braziliensis/T.Cruzi*
**V61**	*L. braziliensis*	*L. braziliensis/T.Cruzi*
**V62**	*L. braziliensis*	*L. braziliensis/T.Cruzi/L.panamensis*
**V63**	*L. braziliensis*	*L. braziliensis/T.Cruzi*
**V64**	*L. braziliensis*	*L. braziliensis/T.Cruzi*
**MT43**	*T. cruzi*	*T.cruzi/L.panamensis*
**MT53**	*T. cruzi*	*T. cruzi*
**MT56**	*T. cruzi*	*T. cruzi*
**MT59**	*T. cruzi*	*T. cruzi/L.braziliensis/L.panamensis*
**MT63**	*T. cruzi*	*T. cruzi*
**MT83**	*T. cruzi*	*T. cruzi/L.panamensis*
**R94**	*L. panamensis*	*L.panamensis/L.braziliensis/L.naiffi*
**R95**	*L. braziliensis*	*L.panamensis/L.braziliensis/L.naiffi*

Table S4: Sequencing statistics of Amplicon-base NGS for each sample included in the study.

Sample ID	Sequencing depth	q20_reads (QC)	% reads pass QC	blast_reads	% reads taxonomically assigned by BLASTn
BAS 03	166244	145447	87.5	144376	99.3
BAS 06	162415	148508	91.4	147912	99.6
BAS 10	168983	159950	94.7	159819	99.9
BAS 112	174314	154307	88.5	146919	95.2
BAS 114	171460	150414	87.7	135522	90.1
BAS 126	164459	153572	93.4	150462	98.0
BAS 19	160671	152821	95.1	152545	99.8
BAS 31	163443	151081	92.4	150884	99.9
BON 100	167370	153007	91.4	134826	88.1
BON 12	165228	156538	94.7	138362	88.4
BON 15	164625	145504	88.4	143742	98.8
BON 18	161017	140726	87.4	133320	94.7
BON 21	173351	163406	94.3	162371	99.4
BON 22	162257	142866	88.0	142543	99.8
BON 32B	167157	158608	94.9	157979	99.6
BON 63	171252	155862	91.0	153758	98.7
BON 75	161420	150885	93.5	150214	99.6
BON 83	160200	150501	93.9	148544	98.7
BON 91	168020	159696	95.0	159269	99.7
BON 92	175133	161879	92.4	155759	96.2
BON 94	172535	162564	94.2	116140	71.4
BON 95	178350	168111	94.3	165863	98.7
H88	164735	151262	91.8	150931	99.8
H89	178035	165547	93.0	165338	99.9
P29	167558	153756	91.8	153688	100.0
P4	164154	155482	94.7	154263	99.2
P48A	163185	150373	92.1	150287	99.9
P49	165565	148541	89.7	148331	99.9
P50	167778	153517	91.5	153031	99.7
P52	174596	161896	92.7	161540	99.8
P55	163673	154832	94.6	153767	99.3
P56	177887	169587	95.3	169086	99.7
P57	171316	154749	90.3	150703	97.4
P58	179328	163492	91.2	163324	99.9
P59	165257	151038	91.4	150735	99.8
P61	163811	150356	91.8	150189	99.9
P64	175513	157668	89.8	156448	99.2
P69	175492	165401	94.2	162024	98.0
P70	177725	169354	95.3	168536	99.5
P75	161492	153342	95.0	152876	99.7
P77	166105	153576	92.5	152982	99.6
P80	171331	161987	94.5	161871	99.9
P81	166924	159001	95.3	153001	96.2
P82	173986	158185	90.9	157189	99.4
P83	170994	156822	91.7	156130	99.6
P84	176278	167734	95.2	167316	99.8
P89	165543	157627	95.2	156992	99.6
P9	173816	163664	94.2	138384	84.6
P99	177196	168378	95.0	168355	100.0
L75	176737	167498	94.8	163345	97.5
V58	170738	159319	93.3	153799	96.5
V59	178315	165414	92.8	150148	90.8

V60	170343	153623	90.2	136839	89.1
V61	178381	167329	93.8	161632	96.6
V62	175509	163376	93.1	144260	88.3
V63	170715	158846	93.0	148196	93.3
V64	172125	158690	92.2	154698	97.5
MT43	161238	148234	91.9	130594	88.1
MT53	179347	166222	92.7	164078	98.7
MT56	176922	149492	84.5	89941	60.2
MT59	168054	142679	84.9	122649	86.0
MT63	172149	162236	94.2	162171	100.0
MT83	172382	162718	94.4	161814	99.4
R94	134316	125182	93.2	111431	89.0
R95	165212	69031	41.8	65074	94.3
MX1	161266	143382	88.9	140836	98.2
MX2	166181	139112	83.7	124237	89.3

Table S5: Relative frequency of *Leishmania* species per department.

**Department/Specie**	***L.braziliensis***	***L.panamensis***	***L.braziliensis/L.panamensis***	***L.panamensis/L.braziliensis***	***L.naiffi/L.braziliensis***	***L.naiffi/L.panamensis***	***L.naiffi/L.braziliensis/L.panamensis***	***L.braziliensis/L.panamensis/L.infantum***	***L.lindenbergi/L.braziliensis/L.amazonensis***	***L.mexicana/L.amazonensis/L.infantum/L.braziliensis***
Antioquia	0 (0%)	**1 (100%)**	0 (0%)	0 (0%)	0 (0%)	0 (0%)	0 (0%)	0 (0%)	0 (0%)	0 (0%)
Atlantico	0 (0%)	**1 (100%)**	0 (0%)	0 (0%)	0 (0%)	0 (0%)	0 (0%)	0 (0%)	0 (0%)	0 (0%)
Caqueta	**1 (100%)**	0 (0%)	0 (0%)	0 (0%)	0 (0%)	0 (0%)	0 (0%)	0 (0%)	0 (0%)	0 (0%)
Cauca	0 (0%)	**1 (100%)**	0 (0%)	0 (0%)	0 (0%)	0 (0%)	0 (0%)	0 (0%)	0 (0%)	0 (0%)
Choco	0 (0%)	**1 (100%)**	0 (0%)	0 (0%)	0 (0%)	0 (0%)	0 (0%)	0 (0%)	0 (0%)	0 (0%)
Cundinamarca	0 (0%)	0 (0%)	0 (0%)	**1 (100%)**	0 (0%)	0 (0%)	0 (0%)	0 (0%)	0 (0%)	0 (0%)
Guainia	**3 (100%)**	0 (0%)	0 (0%)	0 (0%)	0 (0%)	0 (0%)	0 (0%)	0 (0%)	0 (0%)	0 (0%)
Guajira	0 (0%)	0 (0%)	0 (0%)	**3 (100%)**	0 (0%)	0 (0%)	0 (0%)	0 (0%)	0 (0%)	0 (0%)
**Guaviare**	**12 (66.6%)**	0 (0%)	**2 (11.1%)**	0 (0%)	**1 (5.5%)**	**1 (5.5%)**	**1 (5.5%)**	0 (0%)	**1 (5.5%)**	0 (0%)
Lara(Venezuela)	0 (0%)	0 (0%)	0 (0%)	0 (0%)	0 (0%)	0 (0%)	0 (0%)	0 (0%)	0 (0%)	**2 (100%)**
Meta	**5 (71%)**	0 (0%)	**2 (29%)**	0 (0%)	0 (0%)	0 (0%)	0 (0%)	0 (0%)	0 (0%)	0 (0%)
Nariño	0 (0%)	**3 (60%)**	**1 (20%)**	0 (0%)	0 (0%)	0 (0%)	0 (0%)	**1 (20%)**	0 (0%)	0 (0%)
Norte de Santander	**2 (100%)**	0 (0%)	0 (0%)	0 (0%)	0 (0%)	0 (0%)	0 (0%)	0 (0%)	0 (0%)	0 (0%)
Putumayo	**1 (100%)**	0 (0%)	0 (0%)	0 (0%)	0 (0%)	0 (0%)	0 (0%)	0 (0%)	0 (0%)	0 (0%)
Santander	0 (0%)	**1 (100%)**	0 (0%)	0 (0%)	0 (0%)	0 (0%)	0 (0%)	0 (0%)	0 (0%)	0 (0%)

Table S6: Shanon and Simpson diversity indices of *Leishmania* species in different hosts and reservoirs. The following table shows the results of the Post-Hoc Test for each comparison for both the Shannon and Simpson indices.

Shannon index

**Comparison**		**Z**	**P.unadj**	**P.adj**
Domestic reservoir	Human	2.7962663	5.17E-03	3.10E-02
Domestic reservoir	*Lutzomyia*	0.6787769	4.97E-01	1.00E+00
Human	*Lutzomyia*	-4.6533158	3.27E-06	1.96E-05
Domestic reservoir	Wild reservoir	2.8061976	5.01E-03	3.01E-02
Human	Wild reservoir	0.6335945	5.26E-01	1.00E+00
Lutzomyia	Wild reservoir	3.5456526	3.92E-04	2.35E-03

Simpson index

**Comparison**		**Z**	**P.unadj**	**P.adj**
Domestic reservoir	Human	2.6053439	9.18E-03	5.51E-02
Domestic reservoir	*Lutzomyia*	0.4666591	6.41E-01	1.00E+00
Human	*Lutzomyia*	-4.728691	2.26E-06	1.36E-05
Domestic reservoir	Wild reservoir	2.6247868	8.67E-03	5.20E-02
Human	Wild reservoir	0.6095699	5.42E-01	1.00E+00
Lutzomyia	Wild reservoir	3.5734254	3.52E-04	2.11E-03

The Kruskall Wallis statistical test was used to identify differences between the groups evaluated. Subsequently, the paired comparison between the groups was performed by means of the Dunn-Bonferroni post-hoc method.

Table S7: Analysis of variance and similarity percentage in communities of *Leishmania* species in different host and reservoirs. The table shows the results of the PERMANOVA statistical test.

Human_Lutzomyia

Species	average	sd	ratio	ava	avb	cumsum	p		% contibution
T. cruzi	0.276851	0.16641	1.6637	0	55.3701	0.3682	0.0002	***	36.82
L. braziliensis	0.269474	0.15631	1.7239	65.787	31.8889	0.7266	0.0094	**	35.84
L. panamensis	0.14702	0.18678	0.7871	25.78	9.0066	0.9221	0.8559		
L. infantum	0.027073	0.09414	0.2876	4.675	0.9186	0.9581	0.7425		
L. amazonensis	0.024889	0.06627	0.3756	2.438	2.8158	0.9912	0.3878		
L. mexicana	0.006594	0.03213	0.2052	1.319	0	1	0.8228		
L. naiffi	0	0	NaN	0	0	1	1		

Lutzomyia _Wild reservoir

Species	average	sd	ratio	ava	avb	cumsum	p		% contibution
T. cruzi	0.223149	0.16743	1.3328	55.3701	100	0.5	0.0702	.	50
L. braziliensis	0.159444	0.13263	1.2022	31.8889	0	0.8573	0.9981		35.73
L. panamensis	0.045033	0.07975	0.5647	9.0066	0	0.9582	0.9929		
L. amazonensis	0.014079	0.04702	0.2994	2.8158	0	0.9897	0.6039		
L. infantum	0.004593	0.01534	0.2994	0.9186	0	1	0.8886		
L. naiffi	0	0	NaN	0	0	1	1		
L. mexicana	0	0	NaN	0	0	1	1		

Human_Domestic reservoir

Species	average	sd	ratio	ava	avb	cumsum	p		% contibution
L. braziliensis	0.306114	0.18804	1.6279	65.787	11.14	0.43	0.0026	**	43
L. panamensis	0.295929	0.13266	2.2308	25.78	72.61	0.8457	0.0488	*	41.57
L. infantum	0.09108	0.07	1.3011	4.675	16.26	0.9736	0.059	.	
L. amazonensis	0.012192	0.05337	0.2284	2.438	0	0.9907	0.5527		
L. mexicana	0.006594	0.03227	0.2043	1.319	0	1	0.5601		
L. naiffi	0	0	NaN	0	0	1	1		
T. cruzi	0	0	NaN	0	0	1	1		

Lutzomyia _Domestic reservoir

Species	average	sd	ratio	ava	avb	cumsum	p		% contibution
L. panamensis	0.318	0.11785	2.6983	9.0066	72.61	0.3863	0.0387	*	38.63
T. cruzi	0.27685	0.16984	1.63	55.3701	0	0.7226	0.0428	*	33.63
L. braziliensis	0.13744	0.11341	1.2119	31.8889	11.14	0.8895	0.99		
L. infantum	0.07687	0.03234	2.3771	0.9186	16.26	0.9829	0.0938	.	
L. amazonensis	0.01408	0.0477	0.2952	2.8158	0	1	0.5171		
L. naiffi	0	0	NaN	0	0	1	1		
L. mexicana	0	0	NaN	0	0	1	1		

Signif. Codes:	0	'***'	0.001	'**'	0.01	'*'	0.05	'.'	0.1
Number of permutation	9999								

In red are highlighting those values with statistically significant, as well as the percentage of contribution evaluated by SIMPER. These analyses are found for each of the comparisons.
